# A Verified ODE Solver and the Lorenz Attractor

**DOI:** 10.1007/s10817-017-9448-y

**Published:** 2018-01-22

**Authors:** Fabian Immler

**Affiliations:** Institut für Informatik, Technische Universität München, Munich, Germany

**Keywords:** Isabelle/HOL, Ordinary differential equation, Rigorous numerics, Poincaré map, Lorenz attractor

## Abstract

A rigorous numerical algorithm, formally verified with Isabelle/HOL, is used to certify the computations that Tucker used to prove chaos for the Lorenz attractor. The verification is based on a formalization of a diverse variety of mathematics and algorithms. Formalized mathematics include ordinary differential equations and Poincaré maps. Algorithms include low level approximation schemes based on Runge–Kutta methods and affine arithmetic. On a high level, reachability analysis is guided by static hybridization and adaptive step-size control and splitting. The algorithms are systematically refined towards an implementation that can be executed on Tucker’s original input data.

## Introduction

Computer assisted proofs, i.e., mathematical proofs that rely on the output of a computer program, depend crucially on the correctness of the program. Important computer assisted proofs for e.g., the Kepler conjecture or the Four Color Theorem, have therefore been formally verified. In this article, we consider the Lorenz attractor—perhaps one of the most prominent examples of deterministic chaos—and its computer assisted proof by Warwick Tucker. The proof relies on a rigorous numerical ordinary differential equation (ODE) solver. In this article, we describe the long-term project of formally verifying (in Isabelle/HOL [39]) an ODE solver that is capable of certifying Tucker’s computations.

### History

In 1963, meteorologist Edward Lorenz [31] introduced a system of ODEs as a simplified model for atmospheric dynamics. He observed that even the smallest perturbation in initial values would lead to completely different long-term behavior of the system. Referring to the original motivation, he asked: “Does the Flap of a Butterfly’s Wings in Brazil Set Off a Tornado in Texas?” and the term *butterfly effect* entered popular culture. The Lorenz system tends to evolve to a complicated structure (Fig. 2), which became an iconic example of deterministic chaos: According to Sparrow [45] “the number of man, woman, and computer hours spent on [the Lorenz equations ...] must be truly immense”. Despite its popularity and the amount of effort put into its study, nobody managed to prove that the Lorenz attractor is chaotic in a rigorous mathematical sense. The problem of rigorously proving chaos in the Lorenz attractor even made it into a list of 18 important problems for the twenty-first century that Field’s medalist Stephen Smale composed in 1998 [43].

Shortly after, Warwick Tucker managed to give an affirmative answer by presenting a computer-assisted proof [47, 48]. Tucker’s programs were written in C${++}$ and are not formally verified. Tucker even discovered (and fixed) some bugs in it [46, 49]. Formal verification of the numerical results needed for the proof is therefore a worthwhile goal.

### The Lorenz Attractor

We start with describing the Lorenz attractor and some of the properties that were conjectured from numerical simulations. In his proof, Tucker considers the following three dimensional ODE^1^ for fixed parameters $k_{1,2,3}, \lambda _{1,2,3}$:

$$\begin{aligned} \dot{x}&= \lambda _1 x - k_1(x + y)z \\ \dot{y}&= \lambda _2 y + k_1(x + y)z \\ \dot{z}&= \lambda _3 z + (x + y)(k_2 x + k_3 y) \end{aligned}$$

As intuition, an ODE describes the velocity vector $(\dot{x}, \dot{y}, \dot{z})$ in which a particle at a point (*x*, *y*, *z*) moves. The evolution of a particle subject to the ODE is described by the so-called *flow* $\phi$. A particle $x_0 \in \mathbb {R}^{n}$ will be at position $\phi (x_0, t)$ after time $t \in \mathbb {R}$.

**Fig. 1:**
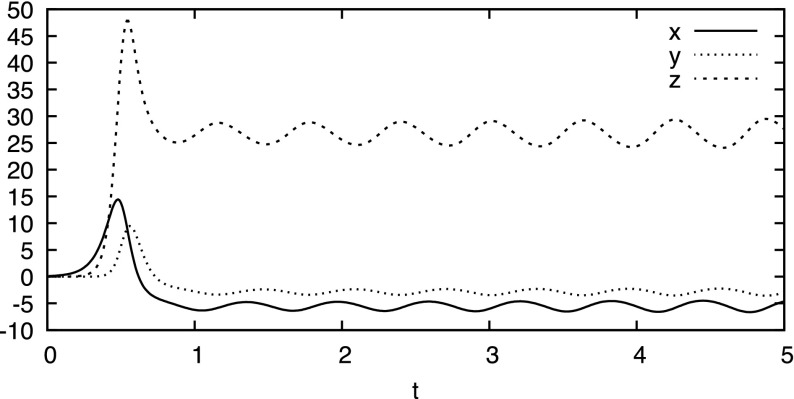
Temporal evolution of $t \mapsto \phi ((0.1, 0, 0), t)$

**Fig. 2:**
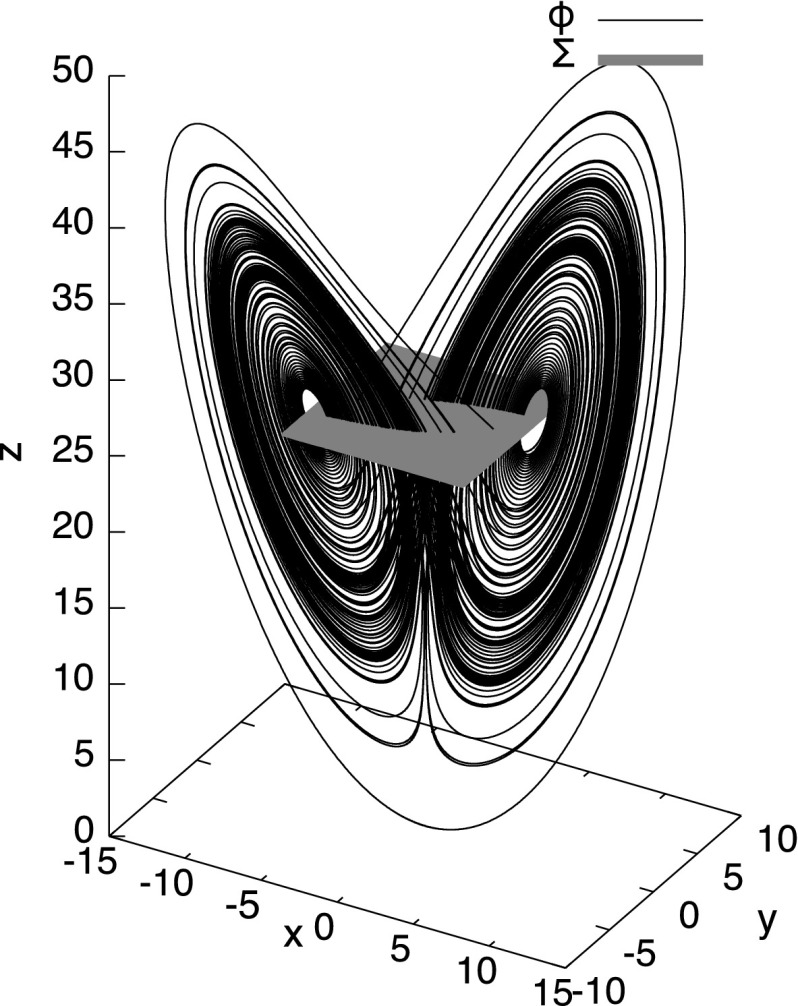
Simulation of a part of the Lorenz attractor ($\phi$) and Poincaré section (${\varSigma }$)

Figure 1 depicts the numerical simulation of the evolution of a particle starting at (0.1, 0, 0): It moves to right ($x \approx 15$) and up ($z \approx 50$) at time $t\approx 0.5$, then down to about $z = 27$ and oscillates with increasingly larger amplitude around $z=27$. Figure 2 depicts the trace of a long-term evolution in the three dimensional phase space, it indicates Property 1:

#### Property 1

Solutions remain in a bounded region of the phase space.

Particles that approach the origin (0, 0, 0) from above exhibit a very sensitive dependence on initial conditions: a slight perturbation can make the particle flow to either the left or right branch of the Lorenz attractor, which we call Property 2:

#### Property 2

Solutions exhibit sensitive dependence on initial conditions.

This dependence is such that arbitrarily small initial sets will eventually spread over the whole attractor.

### Tucker’s Proof

How does Tucker go about proving those properties? First of all, he uses a standard technique: he introduces a so-called Poincaré section. This is a distinguished set in the phase space, in this case a square on the plane $z = 27$, namely ${\varSigma }= [-6, 6] \times [6, 6] \times \{27\}$. Compare also Fig. 2.

On a Poincaré section ${\varSigma }$, one defines the so-called Poincaré map *P*: For a particle $x_0 \in {\varSigma }$, the Poincaré map $P(x_0)$ is the point where the flow first returns to ${\varSigma }$. This reduces the three-dimensional, continuous dynamics $\phi$ to (discrete) iterations of the two-dimensional map *P*. Tucker then analyzes the dynamics of *P*.

*Trapping Region* Regarding Property 1, Tucker proves that there is a (compact) trapping region $N \subseteq {\varSigma }$, such that solutions starting in *N* will remain in *N*. He does so by subdividing *N* into a large number of small rectangles. For every small rectangle, Tucker’s program computes safe numeric bounds for all solutions evolving from the small rectangle. In a number of time-discretization steps, the evolution is followed until it eventually returns to ${\varSigma }$. Upon return, the program checks that the returned enclosure is contained in *N*. If this process succeeds for every small rectangle, one can conclude the following theorem.

#### Theorem 1

(Trapping Region) $$ \forall x \in N - {\varGamma }.~P(x) \in N $$

Note that there exists a set ${\varGamma }$ on which *P* is not defined: ${\varGamma }$ is the set of points, from which solutions tend to the origin in infinite time. ${\varGamma }$ is therefore explicitly excluded in the above theorem.

*Sensitive Dependence* Regarding Property 2, sensitive dependence on initial conditions can be quantified with the help of the derivative: A deviation in the direction of a vector $v \in \mathbb {R}^{2}$ is propagated (in linear approximation) like the derivative at *x*, i.e., $P(x + v) \approx x + \mathsf {D}P|_{x}\cdot v$. Here $\mathsf {D}P|_{x}\cdot v$ is the matrix of partial derivatives of *P* (the Jacobian matrix) at the point *x*, multiplied with the vector *v*.

A mathematically precise notion of chaos is given by the class of singular hyperbolic systems [36]. The term singular denotes the special case where the system contains a hyperbolic fixed point (which renders *P* undefined on ${\varGamma }$). A hyperbolic system contracts deviations in *stable* directions and expands deviations in *unstable* directions. Both are relevant for the dynamics of the attractor: Stable directions make solutions tend to the attractor, whereas unstable directions lead to sensitive dependence on initial conditions.

**Fig. 3:**
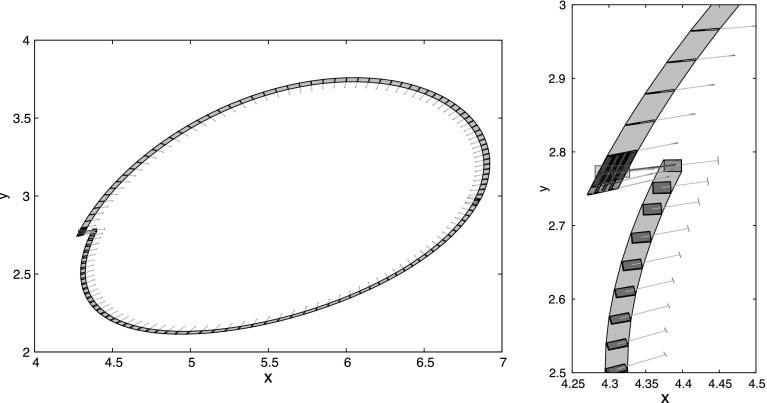
Enclosures for the flow and cones evolving from $X_0 = [4.375, 4.4] \times [2.77, 2.79] \times \{27\}$ with a representation of a cone between $1.5^{\circ }$ and $11.5^{\circ }$ (detail on the right)

Tucker proves that the Lorenz attractor is hyperbolic (in fact singular hyperbolic, we discuss how the hyperbolic fixed point is addressed with normal form theory in the next paragraph) by providing safe overapproximations for the unstable direction: Every $x \in N$ is equipped with a cone $\mathfrak {C}(x)$ (compare Fig. 3), which contains the unstable direction. This is also verified by Tucker’s computer program: In addition to the Poincaré map, the program keeps bounds on its matrix of partial derivatives. The program tracks how initial deviations (inside the cone associated to an initial rectangle) are propagated by the derivative $\mathsf {D}P$. The cone field needs to be forward invariant (otherwise it would not contain the unstable direction) and the expansion needs to be large enough that the enclosed directions are actually expanding. Tucker’s program establishes factors $\mathcal {E}(x)$ and $\mathcal {E}^{-1}(x)$, which quantify the expansion properties of *P*:

#### Theorem 2

(Derivatives, Cones, and Expansion)

1. $$\forall x \in N - {\varGamma }.~\forall v \in \mathfrak {C}(x).~\mathsf {D}P|_{x}\cdot v \in \mathfrak {C}(P(x))$$
2. $$\forall x \in N - {\varGamma }.~\forall v \in \mathfrak {C}(x).~\left\| \mathsf {D}P|_{x}\cdot v\right\| \ge \mathcal {E}(x)\left| v\right| $$
3. $$\forall x \in N - {\varGamma }.~\forall v \in \mathfrak {C}(x).~\left\| \mathsf {D}P|_{x}\cdot v\right\| \ge \mathcal {E}^{-1}(P(x))\left\| v\right\| $$

This theorem states that 1., the cone field $\mathfrak {C}$ is forward invariant under the action of the derivative of *P*: the image of every cone is slimmer than the cones onto which they are mapped. 2., the vectors *v* satisfy lower bounds on how much they are expanded: the length $\left\| \mathsf {D}P|_{x}\cdot v\right\|$ of the return of the deviation vector *v* is lower bounded by its length $\Vert v\Vert$ times an expansion factor $\mathcal {E}(x)$. They also satisfy a pre-expansion bound $\mathcal {E}^{-1}(x)$ (this does not denote $\frac{1}{\mathcal {E}}$) for the pre-image of *x*, which is required for technical reasons in Tucker’s proof.

*Normal Form Theory* For the Lorenz equations, the origin (0, 0, 0) is a hyperbolic fixed point. The origin is a fixed point, because the ODE evaluates to 0. It is hyperbolic, because solutions tend to it in the two stable directions given by the *y* and *z* axis and expands in the unstable direction given by the *x*-axis.

This hyperbolic fixed point poses problems for the aforementioned approach of using rigorous numerical methods: there are solutions that tend to the origin as time goes to infinity. In such a situation, a time-discretization algorithm is at a loss, because it would need infinitely many steps. To remedy this problem, Tucker’s program interrupts computations in a small cube $L = [-0.1, 0.1] \times [-0.1, 0.1] \times [-0.1, 0.1]$ around the origin. Inside the cube, where the numerical methods would fail, the evolution of solutions can be described with classical, analytical means: more than half of Tucker’s thesis is devoted to accurate analytical expressions for the flow inside the cube *L*. These expressions can be used to provide explicit bounds on how solutions exit the cube *L* and continue with numerical computations.

#### Informal Theorem 3

There is an explicit form that bounds the dynamics inside the cube $L = [-0.1, 0.1] \times [-0.1, 0.1] \times [-0.1, 0.1]$.

### Outline of This Article

This article is about the formalization of a rigorous ODE solver that computes the Poincaré map *P* and its derivative $\mathsf {D}P$. It is sufficiently efficient and precise to certify Tucker’s numerical results. In particular, computing with the verified algorithm proves Theorems 1 and 2. In fact, also Fig. 3 was created from the output of this verified algorithm.

As a matter of course, since the ODE solver computes Poincaré maps and derivatives thereof, it is proved correct with regard to a formalization of these concepts in Isabelle/HOL. This formalization, as well as the verified algorithms are generic: they are independent of the underlying ODE or dimension.

This article summarizes previous work of the author (and contributions by Johannes Hölzl and Christoph Traut) [18–26] and describes the state of the formalization to which it evolved over time. It shows how the various parts fit together for the final result of certifying Tucker’s computations. The following explains how this article is structured and details on the relation to earlier publications.

The abstract mathematics needed for the formalization is a theory of ODEs and Poincaré maps together with their (differentiable) dependence on initial conditions. This is presented in Sect. 2, which summarizes a more comprehensive journal article [25], in which the author extends the conference paper [26] with a formalization of Poincaré maps. The foundations, in particular existence and uniqueness of solutions was proved in [24].

Tucker used the library Profil/BIAS for an implementation of the Euler method in interval arithmetic. Our approach to rigorous numerics is at first agnostic about the concrete type of enclosures (Sect. 3). The main instantiation, affine arithmetic, is presented in Sect. 3.4. Parts of this section were part of earlier publications [18, 21].

When working with affine arithmetic, the enclosures are zonotopes (centrally symmetric polytopes) instead of intervals. Because zonotopes are a more complex than intervals, geometric operations like intersections with the Poincaré section ${\varSigma }$ are more challenging. The verification leads to a digression into computational geometry in Sect. 4, which is based on an earlier publication [19].

Tucker’s program adaptively splits reachable sets and therefore maintains a collection of sets. Section 5 describes how we formalize generic data structures to maintain such collections and use stepwise refinement from nondeterministic abstract specifications to a concrete deterministic implementation. This has not been published before.

A more ad-hoc formalization of similar algorithms has been described in earlier work [21], which forms the basis of Sect. 6. Here the presentation is w.r.t. the new, generic framework and extended for derivatives of Poincaré maps.

Section 7 is a novel contribution: it describes how the trapping region *N*, the cone field $\mathfrak {C}$ and the expansion estimates $\mathcal {E}, \mathcal {E}^{-1}$ are defined formally and how the verified ODE solver is set up to certify the results of all of Tucker’s computations. Earlier work [20] was not capable of handling derivatives, and had no formalization of Poincaré map.

All of the development described here is available for Isabelle2017 in the Archive of Formal Proof [22, 23]. In particular everything displayed as **Theorem** possesses a formal counterpart.

## Mathematics

The required mathematical background consists of mostly standard results which can be found in every textbook on ODEs and dynamical systems. Thanks to a sufficient background theory, the formalization can mostly follow the presentations in such textbooks. We therefore focus on peculiarities of our formalization which are due to Isabelle/HOL and its type system, in particular the use of type classes and the lack of dependent types. Because of the type class based formalization of topological structures, type definitions are used to formalize function spaces, where the Transfer and Lifting tools [15] provide excellent support. Moreover, there are no dependent types in Isabelle/HOL. In situations where this would be more natural, an encoding (e.g., ${{\textsf {\textit{ext-cont}}}}\,$ in the following Sect. 2.1.2) is necessary.

### Type Classes for Mathematics in Isabelle/HOL

In Isabelle/HOL, many of the mathematical concepts (in particular spaces with a certain structure) are formalized using type classes. Isabelle/HOL features axiomatic type classes [11, 38]. The purpose of an axiomatic type class is to specify operations which satisfy given properties for a class of types. The advantage of type class based reasoning is that most of the reasoning is generic: formalizations are carried out in the context of type classes and can then be used for all types inhabiting that type class.

For generic formalizations, we use Greek letters $\alpha , \beta , \gamma$ and name their type class constraints in prose. I.e., when we write that we “consider a topological space” $\alpha$, then this result is formalized generically for every type $\alpha$ that fulfills the properties of a topological space.

The spaces we consider are topological spaces with ${{\textsf {\textit{open}}}}\,$ sets, (real) vector spaces with addition $+: \alpha \rightarrow \alpha \rightarrow \alpha$ and scalar multiplication $(\_)(\_): \mathbb {R}\rightarrow \alpha \rightarrow \alpha$. Normed vector spaces come with a norm $|(\_)| : \alpha \rightarrow \mathbb {R}$. Complete normed vector spaces are called Banach spaces. Much of the theory has been ported from Harrison’s theory of Euclidean space [12] and has been generalized to the hierarchy of type classes for mathematics in Isablle/HOL [14].

#### Vectors in Euclidean Space

Because of Isabelle/HOL’s restrictive type system (no dependent types), the abstract concept of vectors is notorious for demanding workarounds. In Isabelle/HOL, one tends to use a type class based encoding. We work with a type class for Euclidean space that fixes an order on the Basis elements and therefore enables operations ${{\textsf {\textit{eucl-of-list}}}}\,:\mathbb {R}\,{{\textsf {\textit{list}}}}\,\rightarrow \alpha$ and ${{\textsf {\textit{list-of-eucl}}}}\,:\alpha \rightarrow \mathbb {R}\,{{\textsf {\textit{list}}}}\,$ if $\alpha$ is a Euclidean space.

All (finite) vectors of real numbers are instances of the class Euclidean space. This includes real numbers $\mathbb {R}$, complex numbers $\mathbb {C}$, tuples $\alpha \times \beta$ for Euclidean spaces $\alpha , \beta$, and Harrison-style^2^ [12] vectors $\alpha ^\iota$ for a finite type $\iota$.

#### Bounded Continuous Function

We motivate bounded continuous functions with the Picard–Lindelöf theorem, which guarantees the existence of a unique solution to an initial value problem. For an ODE *f* with initial value $x_0$ at time $t_0$, a unique solution on the time interval $[t_0, t_1]$ is constructed by considering iterations of the following operator for continuous functions $\phi : [t_0, t_1] \rightarrow \mathbb {R}^{n}$:

$$\begin{aligned} P(\phi ) {:}= \left( \lambda t.~x_0 + \int _{t_0}^{t} f(\tau , \phi (t)) \,\mathrm {d}\tau \right) \end{aligned}$$

From a mathematician’s point of view, *P* operates on the Banach space of continuous functions on the compact domain $[t_0, t_1]$ and therefore the Banach fixed point theorem guarantees the existence of a unique fixed point (which is by construction the unique solution).

In order to formalize this in Isabelle/HOL, there are two obstructions to overcome: First, the concept of Banach space is a type class in Isabelle/HOL, so we need to introduce a type for the mappings $$\phi : [t_0, t_1] \rightarrow \mathbb {R}^{n}$$ from above. But this poses the second problem: functions in Isabelle/HOL are total and types must not depend on term parameters like $$t_0$$ and $$ t_1$$.

We work around these restrictions by introducing a type of *bounded* continuous functions, which is a Banach space and comprises (with a suitable choice of representations) all continuous functions on all compact domains.

$$\begin{aligned} {\mathbf {\mathsf{{typedef}}}}~\alpha \rightarrow _{{{\textsf {\textit{bc}}}}\,}\beta {:}= \{f:\mathbb {R}^{n} \rightarrow \mathbb {R}^{m} \mid f~\text {continuous on}~\alpha \wedge (\exists B.~\forall t.~\Vert f~t\Vert \le B)\} \end{aligned}$$

In order to define operations on type $\alpha \rightarrow _{{{\textsf {\textit{bc}}}}\,}\beta$, the Lifting and Transfer package [15] is an essential tool: operations on the plain function type $\alpha \rightarrow \beta$ are automatically lifted to definitions on the type $\alpha \rightarrow _{{{\textsf {\textit{bc}}}}\,}\beta$ when supplied with a proof that functions in the result are bounded continuous under the assumption that argument functions are bounded continuous. We write application $\;\$_{{{\textsf {\textit{bc}}}}\,}\;$ of a bounded continuous function $f:\alpha \rightarrow _{{{\textsf {\textit{bc}}}}\,}\beta$ to an element $x:\alpha$ as follows.

##### Definition 1

(*Application of Bounded Continuous Functions*)

$$\begin{aligned} (f \;\$_{{{\textsf {\textit{bc}}}}\,}\;x) : \beta \end{aligned}$$

Bounded continuous functions form a normed vector space. The norm on $\alpha \rightarrow _{{{\textsf {\textit{bc}}}}\,}\beta$ is the supremum of the range and the vector space operations ${+}$, ${\cdot }$ are defined pointwise.

##### Definition 2

(*Normed Vector Space of Bounded Continuous Functions*)

$$\begin{aligned} \begin{array}{rcl} \Vert f\Vert &{}{:}=&{} \sup ~\{\Vert f\;\$_{{{\textsf {\textit{bc}}}}\,}\;x\Vert \mid x \in \alpha \}\\ (f + g)\;\$_{{{\textsf {\textit{bc}}}}\,}\;x &{}{:}=&{} f \;\$_{{{\textsf {\textit{bc}}}}\,}\;x + g \;\$_{{{\textsf {\textit{bc}}}}\,}\;x\\ (a \cdot f)\;\$_{{{\textsf {\textit{bc}}}}\,}\;x &{}{:}=&{} a \cdot (f \;\$_{{{\textsf {\textit{bc}}}}\,}\;x))\\ \end{array} \end{aligned}$$

The type $\rightarrow _{{{\textsf {\textit{bc}}}}\,}$ with the above operations forms a complete normed vector space (a Banach space). This allows us to use the Banach fixed point theorem for operators on this type.

In order to be able to use this for the operator *P* from above, we represent functions on a compact interval [*a*, *b*] as an element of type $\rightarrow _{{{\textsf {\textit{bc}}}}\,}$ by extending the function continuously outside the domain with the help of ${{\textsf {\textit{clamp}}}}\,$:

$$\begin{aligned} \begin{array}{rcl} {{\textsf {\textit{clamp}}}}\,_{[a, b]}~x &{} {:}= &{} {\mathbf {\mathsf{{if}}}}~x \le a~{\mathbf {\mathsf{{then}}}}~a~{\mathbf {\mathsf{{else}}}}~({\mathbf {\mathsf{{if}}}}~x \ge b~ {\mathbf {\mathsf{{then}}}}~b~{\mathbf {\mathsf{{else}}}}~x) \\ ({{\textsf {\textit{ext-cont}}}}\,_{[a, b]}~f)\;\$_{{{\textsf {\textit{bc}}}}\,}\;x &{} {:}= &{} f~({{\textsf {\textit{clamp}}}}\,_{[a, b]}~x)) \\ \end{array} \end{aligned}$$

With the help of ${{\textsf {\textit{ext-cont}}}}\,$ we can apply $P:(\mathbb {R}\rightarrow _{{{\textsf {\textit{bc}}}}\,}\mathbb {R}^{n})\rightarrow (\mathbb {R}\rightarrow _{{{\textsf {\textit{bc}}}}\,}\mathbb {R}^{n})$ to a continuous function $\phi : \mathbb {R}\rightarrow \mathbb {R}^{n}$ (that is assumed to be continuous on an interval [*a*, *b*]) by writing $P ({{\textsf {\textit{ext-cont}}}}\,_{[t_0, t_1]} \phi )$. According to the Banach fixed point theorem there exists a unique fixed point $\phi _{bc}:\mathbb {R}\rightarrow _{{{\textsf {\textit{bc}}}}\,}\mathbb {R}^{n}$ where $P(\phi _{bc}) = \phi _{bc}$ and the unique solution of the initial value problem is the function $\lambda t.~\phi _{bc} \;\$_{{{\textsf {\textit{bc}}}}\,}\;t$ of type $\mathbb {R}\rightarrow \mathbb {R}^{n}$.

The usage of the type $\rightarrow _{{{\textsf {\textit{bc}}}}\,}$ caused minor technical obstructions, but otherwise enabled a natural and abstract proof.

#### Bounded Linear Functions

Similar to the type of bounded continuous functions, we also introduce a type of bounded *linear* functions (also known as continuous linear functions)

For vector spaces $\alpha$ and $\beta$, a linear function is a function $f : \alpha \rightarrow \beta$ that is compatible with addition and scalar multiplication.

$$\begin{aligned} {{\textsf {\textit{linear}}}}\,~f {:}= \forall x\,y\,c.~f(c \cdot x + y) = c\cdot f(x) + f(y) \end{aligned}$$

Let us assume normed vector spaces $\alpha$ and $\beta$. Linear functions are continuous if the norm of the result is linearly bounded by the norm of the argument. We cast bounded linear functions $\alpha \rightarrow \beta$ as a type $\alpha \rightarrow _{{{\textsf {\textit{bl}}}}\,}\beta$ in order to make it an instance of Banach space.

$$\begin{aligned} {\mathbf {\mathsf{{typedef}}}}~\alpha \rightarrow _{{{\textsf {\textit{bl}}}}\,}\beta {:}= \left\{ f:\alpha \rightarrow \beta \mid {{\textsf {\textit{linear}}}}\,~f \wedge \exists K.~\forall x.~\Vert f(x)\Vert \le K \Vert x\Vert \right\} \end{aligned}$$

The construction is very similar to bounded continuous functions and we write bounded linear function application $(f \cdot _{{{\textsf {\textit{bl}}}}\,}x)$. Vector space operations are also analogous to $\rightarrow _{{{\textsf {\textit{bc}}}}\,}$. The usual choice of a norm for bounded linear functions is the operator norm: the maximum of the image of the bounded linear function on the unit ball. With this norm, $\alpha \rightarrow _{{{\textsf {\textit{bl}}}}\,}\beta$ forms a normed vector space and we prove that it is Banach if $\alpha$ is a normed vector space and $\beta$ is Banach.

##### Definition 3

(*Norm in Banach Space* ${\varvec{\rightarrow }}_{{\varvec{\mathsf{{ bl}}}}}$) For $f:\alpha \rightarrow _{{{\textsf {\textit{bl}}}}\,}\beta$,

$$\begin{aligned} \Vert f\Vert {:}= {{\textsf {\textit{onorm}}}}\,(\lambda y.~f \cdot _{{{\textsf {\textit{bl}}}}\,}y) = \max \left\{ \Vert f \cdot _{{{\textsf {\textit{bl}}}}\,}y\Vert \mid \Vert y\Vert \le 1 \right\} \end{aligned}$$

Having (bounded) linear functions as a separate type makes many formulations easier. For example, consider Harrison’s formalization of multivariate analysis (from which Isabelle/HOL’s analysis descended). In Harrison’s formalization continuity is formalized for functions *f* of type $\mathbb {R}^{n}\rightarrow \mathbb {R}^{m}$.

$$\begin{aligned} ({{\textsf {\textit{continuous}}}}\,~f~({{\textsf {\textit{at}}}}\,~x)) = (\forall e>0.~\exists d>0.~\forall y.~\Vert x - y\Vert< d \implies \Vert f~x - f~y\Vert < e) \end{aligned}$$

Most of Harrison’s formalization is geared towards viewing derivatives as linear functions of type $\mathbb {R}^{n} \rightarrow \mathbb {R}^{m}$. For continuously differentiable functions, one therefore needs to reason about functions $f' : \mathbb {R}^{n} \rightarrow (\mathbb {R}^{n} \rightarrow \mathbb {R}^{m})$, where $f'~x$ is the derivative of *f* at a point *x*. Continuity of $f'$ is written in an explicit $\varepsilon$-$\delta$ form and involves the operator norm ${{\textsf {\textit{onorm}}}}\,: (\mathbb {R}^{n}\rightarrow \mathbb {R}^{m})\rightarrow \mathbb {R}$, which is quite verbose:

$$\begin{aligned} (\forall e>0.~\exists d>0.~\forall y.~|x - y|< d \implies {{\textsf {\textit{onorm}}}}\,(\lambda v.~f'~x~v - f'~y~v) < e)) \end{aligned}$$

The $\varepsilon$-$\delta$ form could of course be captured in a separate definition, but this would be very similar to the definition of continuity and would introduce redundancy.

In the Isabelle/HOL formalization, ${{\textsf {\textit{continuous}}}}\,$ is defined for functions $f : \alpha \rightarrow \beta$ for topological spaces $\alpha$ and $\beta$. If $\alpha$ and $\beta$ are normed vector spaces, the above equality for ${{\textsf {\textit{continuous}}}}\,$ holds in Isabelle/HOL, too. And indeed, the norm of bounded linear functions is defined using ${{\textsf {\textit{onorm}}}}\,$ such that ${{\textsf {\textit{onorm}}}}\,(\lambda v.~(f'~x)\cdot _{{{\textsf {\textit{bl}}}}\,}v - (f'~y) \cdot _{{{\textsf {\textit{bl}}}}\,}v) = \Vert f' x - f' y\Vert$ holds. Then, continuity of a derivative $f' : \alpha \rightarrow (\alpha \rightarrow _{{{\textsf {\textit{bl}}}}\,}\beta )$ can simply be written as $({{\textsf {\textit{continuous}}}}\,~f'~({{\textsf {\textit{at}}}}\,~x))$, which is a better abstraction to work with and also avoids redundant formalizations for different kinds of continuity.

### Dynamical Systems

An ODE induces a continuous dynamical system via the notion of flow. A standard technique to reason about such systems is its Poincaré map. We keep the presentation at a high level, since details can be found in the publications [25, 26].

#### The Flow

We consider an autonomous ODE with right hand side *f*. Under mild assumptions, there exists a solution $\phi (t)$, which is unique for an initial condition $x(0) = x_0$ and satisfies the differential equation:

$$\begin{aligned} \dot{x}(t) = f(x(t)) \end{aligned}$$

To emphasize the dependence on the initial condition, one writes $\phi (x_0, t)$ for the solution. This solution depending on initial conditions is called the *flow* of the differential equation:

##### Definition 4

(*Flow*) The *flow* $\phi (x_0, t)$ is the (unique) solution of the ODE $\dot{x}(t) = f(x(t))$ with initial condition $\phi (0) = x_0$

The flow is only well-defined on the so-called existence interval of the solution, which depends on the initial value.

##### Definition 5

(*Existence Interval*) $$t \in {{\textsf {\textit{ex-ivl}}}}\,(x_0) \implies \dot{\phi }(x_0, t) = f(\phi (x_0, t))$$

The flow $\phi$ and the existence interval ${{\textsf {\textit{ex-ivl}}}}\,$ provide a clean interface to talk about solutions of ODEs. The property of the generic notion of flow makes it possible to easily state composition of solutions and to algebraically reason about them. Flowing from $x_0$ for time $s + t$ is equivalent to first flowing for time *s*, and from there flowing for time *t*:

##### Theorem 4

(Flow Property)

$$\begin{aligned} \{s, t, s + t\} \subseteq {{\textsf {\textit{ex-ivl}}}}\,(x_0) \implies \phi (x_0, s + t) = \phi (\phi (x_0, s), t) \end{aligned}$$

For Tucker’s proof, one needs to study how sensitive the flow depends on perturbations of the initial value. We use two main results: One, the flow depends continuously on initial values. Two, if the ODE *f* is continuously differentiable, then so is the flow. We first take a look at the domain ${\varOmega }= \left\{ (x, t)\mid ~t \in {{\textsf {\textit{ex-ivl}}}}\,(x)\right\} \subseteq X \times T$ of the flow. $(t, x) \in {\varOmega }$ means that we can flow a point starting at *x* for at least time *t*. Intuitively, solutions starting close to *x* can be followed for times that are close to *t*. In topological parlance, the state space is open.

##### Theorem 5

(Open State Space) ${{\textsf {\textit{open}}}}\,{\varOmega }$

One can show that solutions deviate at most exponentially fast: $\exists K.~\Vert \phi (x, t) - \phi (y, t)\Vert < \Vert x - y\Vert e^{K|t|}$ (using Grönwall’s lemma). Therefore, by choosing *x* and *y* close enough, one can make the distance of the solutions arbitrarily small. In other words, the flow is a continuous function on the state space:

##### Theorem 6

(Continuity of Flow) ${{\textsf {\textit{continuous-on}}}}\,~{\varOmega }~\phi$

Continuity states that small deviations in the initial values result in small deviations of the flow. One can be more precise on the way initial deviations propagate. The propagation of initial deviations through the flow ($\phi _t {:}= \lambda x.~\phi (x, t)$) can be approximated by a linear function, the derivative $\mathsf {D}\phi |_{x} \cdot v \approx \phi (x, t) - \phi (x + v, t)$.

We formalize the fact that the derivative of the flow is the solution of a differential equation in the space of bounded linear mappings, the so-called variational equation.

##### Theorem 7

(Variational Equation)

$$\begin{aligned} {\left\{ \begin{array}{ll} \dot{W}(t) = \mathsf {D}f|_{\phi (x_0, t)} \cdot _{{{\textsf {\textit{bl}}}}\,}W(t)\\ W(0) = 1_{{{\textsf {\textit{bl}}}}\,}\end{array}\right. } \end{aligned}$$

Solving this ODE numerically gives a means to obtain numerical bounds on the derivative, which is the approach that we pursue in our algorithms.

#### The Poincaré Map

The Poincaré map is an important tool for studying dynamical systems. Whereas the flow describes the evolution of a continuous system with respect to *time*, it is the Poincaré map that allows us to study the evolution with respect to some *space* variables. A Poincaré section is a subset ${\varSigma }$ of the state space, which is in general given as an implicit surface ${\varSigma }= \{x \mid s(x) = c\}$ with continuously differentiable *s*. For Tucker’s proof, one chooses $s(x, y, z) = z$ and $c = 27$.

The Poincaré map *P*(*x*) is defined as the point where the flow starting from *x* first hits the Poincaré section ${\varSigma }$. It is defined with the help of the first return time $\tau (x)$. $\tau$ depends on the flow $\phi$ (and therefore on the ODE *f*) and the Poincaré section ${\varSigma }$, but we keep those dependencies implicit.

##### Definition 6

(*First Return Time*) $\tau (x)$ is the least $t>0$ such that $\phi (x, t) \in {\varSigma }$.

Obviously, $\tau$ is only well-defined for values that actually return to ${\varSigma }$, which we encode in the predicate ${{\textsf {\textit{returns-to}}}}\,$:

##### Definition 7

$$\begin{aligned} {{\textsf {\textit{returns-to}}}}\,({\varSigma }, x) {:}= \exists t>0.~\phi (x, t) \in {\varSigma }\end{aligned}$$

The return time can then be used to define the Poincaré map as follows:

##### Definition 8

(*Poincaré map*)

$$\begin{aligned} P(x) {:}= \phi (x, \tau (x)) \end{aligned}$$

It is interesting to note that this way of defining the return time and Poincaré map differs from the usual approach in textbooks. Textbooks study Poincaré maps in a neighborhood around a periodic point $x \in {\varSigma }$, i.e., $P(x) = x$. This makes it easy to directly apply the implicit function theorem and transfer continuity and differentiability from the flow to the Poincaré map while guaranteeing that $\tau$ and *P* are well-defined. Also, one views *P* as a mapping *on* ${\varSigma }$, i.e. $P : {\varSigma }\rightarrow {\varSigma }$.

Tucker’s proof, however, requires a more flexible notion of Poincaré map and our notion of $\tau$ is more flexible: it is well-defined also for values outside of ${\varSigma }$. This enables reasoning about intermediate sections: Tucker, e.g., composes a sequence of local Poincaré maps between intermediate sections ${\varSigma }, {\varSigma }_1, \cdots , {\varSigma }_n, {\varSigma }$ in order to get bounds on the global Poincaré map ${\varSigma }\rightarrow {\varSigma }$.

The goal of Tucker’s computations is a sensitivity analysis of the flow of the Lorenz system and of its Poincaré map. Its derivative can be given in terms of the derivative of the flow and the function *s* defining the implicit surface for ${\varSigma }= \{x \mid s(x) = c\}$.

##### Theorem 8

(Derivative of Poincaré map)

$$\begin{aligned} \mathsf {D}P|_{x}\cdot h = \mathsf {D}\phi |_{(x, \tau (x))}\cdot h - \frac{\mathsf {D}s|_{P(x)}\cdot (\mathsf {D}\phi |_{(x, \tau (x))}\cdot h)}{\mathsf {D}s|_{P(x)}\cdot (f(P(x)))}f(P(x)) \end{aligned}$$

For a rough intuition, the derivative $\mathsf {D}P|_{x}\cdot h$ of the Poincaré map is related to the derivative of the flow $\mathsf {D}\phi |_{(x, \tau (x))}\cdot h$. But it needs to corrected in the direction *f*(*P*(*x*)) in which the flow passes through ${\varSigma }$, because *P* varies only *on* ${\varSigma }$ and not through it. This correction factor also depends on the tangent space $\mathsf {D}s|_{P(x))}$ of the section ${\varSigma }$ at *P*(*x*).

## Rigorous Numerics

**Fig. 4:**
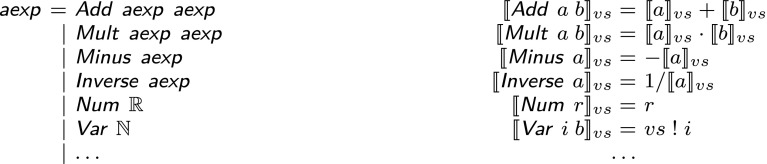
Data type of arithmetic expressions and interpretation function

*Rigorous* (or *guaranteed*) numerics means computing with sets that are guaranteed to enclose the real quantities of interest. Enclosures can in principle be any data structure that represents sets of real values. Popular choices are intervals, zonotopes, or Taylor models. For formalizations, it is useful to have a deep embedding of arithmetic expressions as done, e.g., by Dorel and Melquiond [34] as well as Hölzl [13]. This work builds on Hölzl’s language of arithmetic expressions given in excerpts in Fig. 4.

Independent of the choice of representation of enclosures, a rigorous approximation scheme ${{\textsf {\textit{approx}}}}\,$ needs to satisfy that for all lists of real values *xs* in an enclosure *XS*, the interpretation  of the expression *e* is contained in the result of approximation scheme evaluated for the enclosure *XS*. We could call this the fundamental property of rigorous numerics.The key approach in our formalization is to remain agnostic about the concrete approximation scheme as long as possible and formalize results on the level of deeply embedded ${{\textsf {\textit{aexp}}}}\,$ expressions. The central result is an implementation of a second order Runge–Kutta method on the level of ${{\textsf {\textit{aexp}}}}\,$ expressions. As concrete instance for an approximation scheme ${{\textsf {\textit{approx}}}}\,$, we use affine arithmetic [6], an improvement over interval arithmetic that tracks linear dependencies between program variables.

### Expressions for Vectors

To represent vectors, we use lists of expressions. A list of expressions $es:{{\textsf {\textit{aexp}}}}\,\,{{\textsf {\textit{list}}}}\,$ is interpreted with  componentwise as Euclidean space $\alpha$:In contrast to the interpretation, approximation of a list of expressions should not be componentwise: an approximation function for lists of expressions should be of type ${{\textsf {\textit{approx}}}}\,:{{\textsf {\textit{aexp}}}}\,\,{{\textsf {\textit{list}}}}\,\rightarrow \mathbb {R}\,{{\textsf {\textit{list}}}}\,\,{{\textsf {\textit{set}}}}\,\rightarrow \mathbb {R}\,{{\textsf {\textit{list}}}}\,\,{{\textsf {\textit{set}}}}\,$, which allows ${{\textsf {\textit{approx}}}}\,$ to keep track of dependencies between the components of the result. If the type were e.g., ${{\textsf {\textit{aexp}}}}\,\,{{\textsf {\textit{list}}}}\,\rightarrow \mathbb {R}\,{{\textsf {\textit{set}}}}\,\,{{\textsf {\textit{list}}}}\,\rightarrow \mathbb {R}\,{{\textsf {\textit{set}}}}\,\,{{\textsf {\textit{list}}}}\,$, this could only represent the Cartesian product of the component enclosures.

For a function $f:\mathbb {R}^{n}\rightarrow \mathbb {R}^{m}$, a deep embedding $f_e$ is a list of expressions (of length *m*), that is interpreted over a list of *n* variables.The derivatives with respect to one variable can be computed symbolically from the structure of the expression. This can also be used to compute partial derivatives on the level of expressions. In the multivariate setting, the derivative $\mathsf {D}f|_{x}$ of *f* at *x* is the matrix of its partial derivatives. In general, we can represent matrices as a flat list (according to ${{\textsf {\textit{eucl-of-list}}}}\,$/${{\textsf {\textit{list-of-eucl}}}}\,$ which are also defined for matrices). For computing derivatives, however, we directly produce an expression that is interpreted as the product of the derivative matrix with a vector:$D_e$ takes the derivative with respect to the first *n* variables, and *xs* is assumed to be of length at least *n*. This way, we can produce expressions for higher derivatives:

$$\begin{aligned}&D^0_e (n, f_e, v_e) {:}= f_e \\&D^{i+1}_e (n, f_e, v_e) {:}= D_e (n, D^i_e (n, f_e, v_e), v_e) \end{aligned}$$

Note that the proper interpretation can only be expressed in Isabelle’s type system for fixed values of *i*: the resulting object is an *i*-linear function, so the resulting type depends on a term argument. This could also be encoded as functions taking lists as arguments, but fixed values of *i* suffice for our purposes and we find the interpretation as curried linear mappings more natural. E.g.,


### A Runge–Kutta Method

On the level of expressions, we verified a two-stage Runge–Kutta method ${{\textsf {\textit{rk}}}}\,_h(x) = x + h\cdot \psi _h(x)$, with $\psi _h(x) = (1 - \frac{1}{2p})f(x) + \frac{1}{2p}f(x + hpf(x))$. This Runge–Kutta method ${{\textsf {\textit{rk}}}}\,_h(x_0)$ approximates the solution up to third order: $|\phi (x_0, h) - {{\textsf {\textit{rk}}}}\,_h(x_0)| \in \mathcal {O}(h^3)$. The third order term stems from (multivariate) Taylor series expansions of the solution $\phi$ and the approximation scheme ${{\textsf {\textit{rk}}}}\,_h$. If we set $f' {:}= \lambda x.~\mathsf {D}f|_{x}$ and $f'' {:}= \lambda x.~\mathsf {D}f'|_{x}$, then the remainder is contained in the convex hull of any set that contains ${{\textsf {\textit{rk-remainder}}}}\,_h(s_1, s_2)$ for all $s_1, s_2 \in [0, 1]$.

$$\begin{aligned} \begin{aligned}&{{\textsf {\textit{rk-remainder}}}}\,_h(s_1, s_2) {:}= \frac{h^3}{2}\cdot \left( \frac{1}{3} \big (f''\big (x (hs_1 + t)\big )\right. \\&\left. \qquad \qquad \cdot _{{{\textsf {\textit{bl}}}}\,}\big (f(x (hs_1 + t))\big )\cdot _{{{\textsf {\textit{bl}}}}\,}\big (f(x (hs_1 + t))\big ) \right. \\&\left. \qquad \qquad + f'\big (x (hs_1 + t)\big )\cdot _{{{\textsf {\textit{bl}}}}\,}\big (f'(x (hs_1 + t))\cdot _{{{\textsf {\textit{bl}}}}\,}(f(x (hs_1 + t)))\big ) \right. \\&\left. \qquad - \frac{p}{2}f''\big (x(t) + hps_2f(x(t))\big )\cdot _{{{\textsf {\textit{bl}}}}\,}\big (f(x(t))\big )\cdot _{{{\textsf {\textit{bl}}}}\,}\big (f(x(t))\big ) \right) \end{aligned} \end{aligned}$$

#### Theorem 9

(Runge–Kutta Method with Remainder Term)

$$\begin{aligned} \phi (x_0, h) \in {{\textsf {\textit{rk}}}}\,_h(x_0) + {{\textsf {\textit{convex-hull}}}}\,({{\textsf {\textit{rk-remainder}}}}\,_h([0, 1], [0, 1])) \end{aligned}$$

In order to use this theorem for rigorous numerical computations, we produce a deep embedding of the expressions for ${{\textsf {\textit{rk}}}}\,_h$ and ${{\textsf {\textit{rk-remainder}}}}\,_h$. We do so for an arbitrary ODE $f:\mathbb {R}^{n}\rightarrow \mathbb {R}^{n}$ with deep embedding . Expressions for $f'$ and $f''$ are computed symbolically from $f_e$ via $D^{1,2}_e$ from the previous Sect. 3.1.

### Straight Line Programs

The expression for ${{\textsf {\textit{rk-remainder}}}}\,$ from the previous Sect. 3.2 contains common subexpressions. This is not desirable because one would need to perform redundant computations. We therefore follow Dorel and Melquiond’s [34] approach and use straight-line programs with static single assignment instead of plain expressions.

For us, a straight line program is just a list of arithmetic expressions, which is interpreted according to function ${{\textsf {\textit{slp}}}}\,: {{\textsf {\textit{aexp}}}}\,\,{{\textsf {\textit{list}}}}\, \rightarrow \mathbb {R}\,{{\textsf {\textit{list}}}}\, \rightarrow \mathbb {R}\,{{\textsf {\textit{list}}}}\,$:The idea is that a straight line program only contains unary or binary operations, although this is not required by the definition. The result of the operation is put on top of the evaluation stack. The following example illustrates sharing the term $x + y$ in the expression $(x+y)(x+y)$:

$$\begin{aligned} {{\textsf {\textit{slp}}}}\,~[{{\textsf {\textit{Add}}}}\,({{\textsf {\textit{Var}}}}\,~0) ({{\textsf {\textit{Var}}}}\,~1), {{\textsf {\textit{Mult}}}}\,({{\textsf {\textit{Var}}}}\,~0) ({{\textsf {\textit{Var}}}}\,~0)]~[x, y] = [(x + y)(x + y), x + y, x, y] \end{aligned}$$

We provide a function ${{\textsf {\textit{slp-of}}}}\,$, which eliminates common subexpressions by traversing an expression bottom-up and saving subexpressions in a map that gives the index of the subexpression in the resulting straight line program.

At run-time (this is important to be able to use the ODE solver as a stand-alone tool), in an initialization phase, the ODE solver computes symbolically the derivatives in the expression for ${{\textsf {\textit{rk}}}}\,_h$ and ${{\textsf {\textit{rk-remainder}}}}\,$, does constant propagation (as derivatives can produce 0 constants, this is beneficial) and then compiles the resulting expression with ${{\textsf {\textit{slp-of}}}}\,$ into a straight-line program, which is then used in the course of approximating the ODE in a series of steps.

### Affine Arithmetic

Up to now, we have kept the discussion on the level of expressions, let us now motivate affine arithmetic as a concrete approximation scheme.

The most basic data structure to represent sets is closed intervals $[a, b] = \{ x \mid a \le x \le b \}$, but those suffer from the wrapping effect: rotated boxes cannot be represented without large overapproximations. Moreover dependencies between variables are lost, e.g. for an enclosure $x \in [0, 2]$, the expression $x - x$ evaluates to $[-2, 2]$ in interval arithmetic whereas the exact result would be representable as the interval [0, 0].

Affine arithmetic [6] improves over interval arithmetic by tracking linear dependencies. An affine form *A* is a function where only finitely many arguments map to nonzero values. It is interpreted for a valuation $\varepsilon : \mathbb {N}\rightarrow \mathbb {R}$ :

$$\begin{aligned} {{\textsf {\textit{affine}}}}\,~\varepsilon ~A {:}= A_0 + \sum _i \varepsilon _i A_i \end{aligned}$$

Looking at the interpretation, one often calls the terms $\varepsilon _i$ noise symbols and $A_i$ generators. The idea is that noise symbols are shared between affine forms and that they are treated symbolically, as formal parameters: the sum of two affine forms is given by the pointwise sum of their generators, and multiplication with a constant factor is also done componentwise.

$$\begin{aligned}&{{\textsf {\textit{affine}}}}\,~\varepsilon ~(A + B) {:}= (A_0 + B_0) + \sum _i \varepsilon _i (A_i + B_i)\\&{{\textsf {\textit{affine}}}}\,~\varepsilon ~(c A) {:}= c A_0 + \sum _i \varepsilon _i (c A_i) \end{aligned}$$

The ${{\textsf {\textit{range}}}}\,$ of an affine form is the set of all ${{\textsf {\textit{affine}}}}\,$ evaluations where the noise symbols range over the closed interval $[-1, 1]$. For the range of a list of affine forms, those are evaluated jointly for the same valuation of the noise symbols, reflecting the intuition that those are shared.

$$\begin{aligned}&{{\textsf {\textit{range}}}}\,~A{:}= \left\{ {{\textsf {\textit{affine}}}}\,~\varepsilon ~A \mid \forall i.~-1 \le \varepsilon _i \le 1\right\} \\&{{\textsf {\textit{joint-range}}}}\,~AS{:}= \left\{ {{\textsf {\textit{map}}}}\,~({{\textsf {\textit{affine}}}}\,~\varepsilon )~AS \mid \forall i.~-1 \le \varepsilon _i \le 1\right\} \end{aligned}$$

As a concrete example, let us examine how affine arithmetic handles the dependency problem in the introductory example $x - x$ for $x \in [0, 2]$. The interval [0, 2] is represented by the affine form $1 + 1 \cdot \varepsilon _1$. This is the affine form given by the function $X {:}= (\lambda i.~{\mathbf {\mathsf{{if}}}}~i=0\vee i=1~{\mathbf {\mathsf{{then}}}}~1~{\mathbf {\mathsf{{else}}}}~0)$. For this function, ${{\textsf {\textit{range}}}}\,~X = [0, 2]$ holds. Then, in affine arithmetic, $(1 + 1 \cdot \varepsilon _1) - (1 + 1 \cdot \varepsilon _1) = 0 + 0\cdot \varepsilon _1$, which corresponds to the constant zero function. Therefore ${{\textsf {\textit{range}}}}\,~(X - X) = \{0\}$.

In general, with the help of ${{\textsf {\textit{range}}}}\,$ and ${{\textsf {\textit{joint-range}}}}\,$, we can express correctness of a binary operation like addition e.g., as follows:

$$\begin{aligned}{}[a, b] \in {{\textsf {\textit{joint-range}}}}\,~[A, B] \implies (a + b)\in {{\textsf {\textit{range}}}}\,~(A + B) \end{aligned}$$

Nonlinear operations like multiplication or division are linearized, adding the linearization error as a fresh noise symbol. Consider e.g., multiplication:

$$\begin{aligned} ({{\textsf {\textit{affine}}}}\,~\varepsilon ~A)*({{\textsf {\textit{affine}}}}\,~\varepsilon ~B) = A_0 B_0 + \big (\sum _i \varepsilon _i (A_0 B_i + A_i B_0)\big ) + \big (\sum _{i>0} \varepsilon _i A_i\big )\big (\sum _{i>0} \varepsilon _i B_i\big ) \end{aligned}$$

For a proper valuation with $\varepsilon _i \in [-1, 1]$, the last summand on the right can be bounded by $(\sum _{i>0} |A_i|)(\sum _{i>0} |B_i|)$. Therefore, if *k* is fresh in *A* and *B*, one can set

$$\begin{aligned} {{\textsf {\textit{affine}}}}\,~\varepsilon ~(A*B) {:}= A_0 B_0 + \big (\sum _i \varepsilon _i (A_0 B_i + A_i B_0)\big ) + \varepsilon _k \left( \big (\sum _{i>0} |A_i|\big )\big (\sum _{i>0} |B_i|\big )\right) \end{aligned}$$

and the *k*-th generator bounds the linearization error such that multiplication of affine forms is conservative:

$$\begin{aligned}{}[a, b] \in {{\textsf {\textit{joint-range}}}}\,~[A, B] \implies a*b \in {{\textsf {\textit{range}}}}\,~(A*B) \end{aligned}$$

Similar to the additional noise symbol for a linearization error, also round-off errors can be included as additional noise symbols. We provide affine approximations for the primitive functions listed in Fig. 4. Expressions (and straight line programs) involving these functions can then be approximated by recursively keeping track of the next fresh noise symbols.

During longer computations more and more noise symbols will be added to the affine form, impairing performance in the long run. The number of noise symbols can be reduced by summarizing (or condensing) several noise symbols into a new one. This process discards the correlation mediated by the summarized noise symbols, so a trade-off needs to be found between precision and efficiency. We consider a list of affine forms *AS* and use the notation $AS_i {:}= {{\textsf {\textit{map}}}}\,~(\lambda A.~A_i)~AS$. We call total deviation $|AS|{:}= {{\textsf {\textit{map}}}}\,~(\lambda A.~\sum _i |A_i|)~AS$ the componentwise sum of absolute values. We summarize all symbols *i* with $|AS_i| \le r|AS|$ for a given *summarization threshold* *r*. We found that it is important to perform the above comparison componentwise and not take (like proposed for other implementations of affine arithmetic [6, 42]) the infinity norm on both sides. This is of particular importance when components differ a lot in magnitude.

**Fig. 5:**
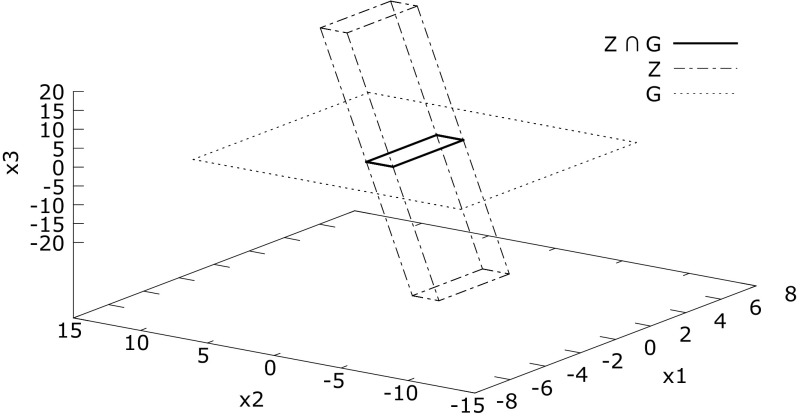
Three dimensional zonotope *Z* and intersection with hyperplane *G*

**Fig. 6:**
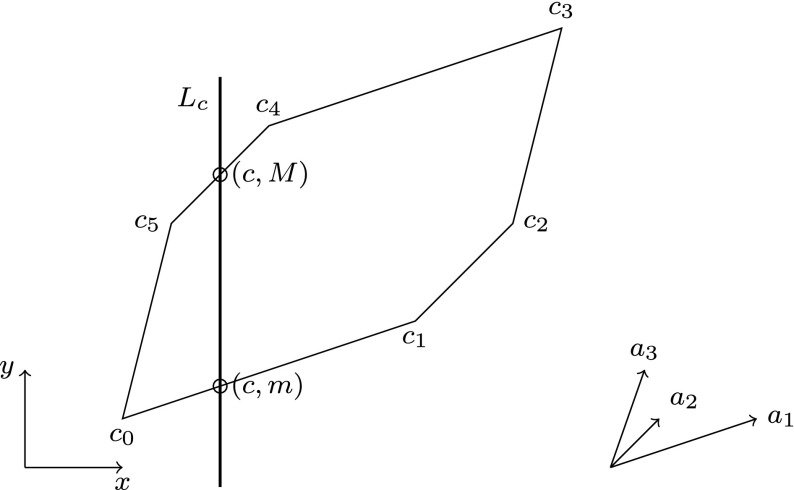
Corners $c_i$ and edges of a zonotope $\{\sum _i \varepsilon _i \cdot a_i \mid -1 \le \varepsilon _i \le 1\}$, generators $a_1, a_2, a_3$, intersecting line $L_g$

Apart from looking at affine forms as a formal sum, the ${{\textsf {\textit{joint-range}}}}\,$ of a list of affine forms can also be interpreted geometrically as zonotopes: centrally symmetric, convex polytopes. A zonotope can be visualized as the Minkowski sum (the set of all possible sums of elements of two sets $X \oplus Y = \{x + y.~x \in X \wedge y \in Y \}$) of the line segments defined by the generators. For example, Fig. 6 depicts a two-dimensional zonotope with three generators, $[-1, 1]a_1 \oplus [-1, 1]a_2 \oplus [-1, 1] a_3$. Figure 5 contains a three-dimensional zonotope with three generators (a parallelotope), namely the zonotope *Z* defined as follows.

$$\begin{aligned} Z {:}= {{\textsf {\textit{joint-range}}}}\,\left( \begin{array}{c@{\quad }c@{\quad }c} \left[ 1 \varepsilon _1 + 0 \varepsilon _2 + 1\varepsilon _3, \right. \\ 0 \varepsilon _1 + 2 \varepsilon _2 + 5\varepsilon _3, \\ \left. 0 \varepsilon _1 + 0 \varepsilon _2 + 20\varepsilon _3\right] \end{array}\right) \end{aligned}$$

## Computational Geometry

An important step for Tucker’s proof is the reduction to a Poincaré map: intersecting the flow of the ODE with a plane in the state space. In our algorithms, the flow is approximated with affine arithmetic expressions, therefore enclosed by zonotopes.

In order to compute where the flow intersects the hyperplane, one needs to compute the intersection of the enclosing zonotope with the hyperplane (see Fig. 5).

This is an interesting geometric problem and we verified an approximative algorithm due to Girard and Le Guernic [7]. At its core, the algorithm is similar to convex hull computations. We can build on a nice abstraction to reason about it, namely Knuth’s theory of counterclockwise (ccw) systems [27]. We needed, however, to extend Knuth’s theory from discrete to continuous sets of points.

### Girard and Le Guernic’s Algorithm

The complexity for computing the exact intersection of a zonotope with a hyperplane grows exponentially with the number of generators. An overapproximation of the zonotope before computing the intersection is possible but can lead to overly coarse approximations. Therefore Girard and Le Guernic [7] proposed a way to directly compute overapproximations to the intersection.

The first idea is to overapproximate a given set *X* tightly from a set *D* of directions, which can be chosen arbitrarily. For every direction $d \in D \subseteq \mathbb {R}^{n}$, the infimum $m_d$ and supremum $M_d$ of the sets $\{\langle x, d\rangle .~x \in X\}$ needs to be determined ($\langle \_, \_\rangle$ denotes the inner product, also known as the dot product). Geometrically speaking, $m_d$ and $M_d$ give the position of two hyperplanes with normal vector *d*. The two hyperplanes bound *X* from below and above, respectively. An overapproximation *P* is then given by the points between all of these hyperplanes:

$$\begin{aligned} X \subseteq P = \{x \in \mathbb {R}^{n}.~\forall d \in D.~m_d \le \langle x, d\rangle \le M_d \} \end{aligned}$$

The second observation of Girard and Le Guernic is that when the set *X* is the intersection of some set *Z* with a hyperplane $G = \{x.~\langle x, g\rangle = c\}$, then the computation of the overapproximation *P* can be reduced to a two-dimensional problem with the linear transformation ${\varPi }_{g,d}:\mathbb {R}^{n}\rightarrow \mathbb {R}^{2}$, ${\varPi }_{g,d}(x) = (\langle x, g\rangle , \langle x, d\rangle )$.

#### Lemma 1

(Reduction to Dimension Two)

$$\begin{aligned} \{\langle x, d\rangle .~x\in Z \cap G\} = \{ y.~(c,y) \in {\varPi }_{g,d}(Z) \} \end{aligned}$$

This lemma is an easy consequence of the definitions of *G* and ${\varPi }_{g, d}$. For every direction *d*, the theorem allows to reduce the computation of the intersection $Z \cap G$ on the left-hand side to the intersection of the projected two-dimensional zonotope ${\varPi }_{g,d}(Z)$ with the vertical line $L_c = \{(x, y).~x=c\}$.

Computing the intersection of a two-dimensional zonotope like the one given in Fig. 6 and a vertical line $L_c$ can by done by computing bounds on the intersection of the vertical line $L_c$ with every edge. This is easy and intuitive. The more challenging part is to compute the set of edges of a two-dimensional zonotope, which we sketch in the following.

### Computing the Set of Edges.

First of all, one can assume that all generators point upwards. One then starts at the lowest corner ($c_0$ in Fig. 6) and appends to it the “rightmost” generator $a_1$ (twice) to reach $c_1$. One then continues with the “rightmost” of the remaining generators, $a_2$ and is in the process essentially “wrapping up” the hull of the zonotope.

In order to verify such a process, we need a way to reason about “rightmost” vectors (a total order). Similar ideas of “wrapping up” a set of points also occur for convex hull algorithms.

### Knuth’s CCW System

In order to verify geometric algorithms, one needs a formal notion of the geometric concepts involved. For convex hull algorithms, Knuth [27] has given a small theory that axiomatizes the notion of orientation of points. The intuition is that for three points *p*, *q*, *r* in the plane, visiting them in order requires either a counterclockwise (ccw) turn (written *pqr*) or clockwise ($\lnot pqr$) turn. Knuth observed that already few of the properties fulfilled by the ccw predicate *pqr* suffice to define a theory rich enough to formalize many concepts in computational geometry.

The notion of *ccw system* is a set of points together with a ccw predicate written *pqr* for points *p*, *q*, *r*. The ccw predicate needs to satisfy the following properties, inspired by the relations satisfied by points in the plane. For all axioms in the following, there is the additional implicit assumption that the involved points are pairwise distinct. For three points, only simple axioms need to be fulfilled:

- Cyclic Symmetry: $$pqr \implies qrp$$
- Antisymmetry: $$pqr \implies \lnot prq$$
- Nondegeneracy: $$pqr \vee prq$$

**Fig. 7:**
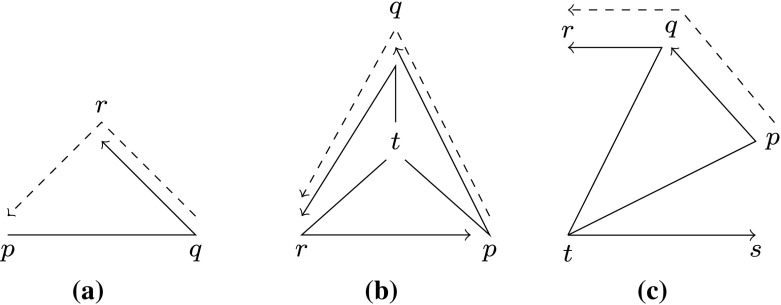
ccw axioms: dashed predicates are implied by solid ones. **a** Cyclic symmetry, **b** interiority, **c** transitivity

Cyclic symmetry and the more interesting case of interiority, which involves four points, are illustrated in Fig. 7. Interiority states that if one point *t* is left of three lines *pq* *qr* *rp*, then the three other points are oriented in a triangle according to *pqr*.

- Interiority: $$tpq \wedge tqr \wedge trp \implies pqr$$

The most important tool for reasoning is transitivity, which involves five points and works if three points *p*, *q*, *r* lie in the half-plane left of the line *ts*, i.e., $tsp \wedge tsq \wedge tsr$. Then, fixing *t* as first element for the ccw relation, we have transitivity in the second and third element: $tpq \wedge tqr \implies tpr$ (see Fig. 7c).

- Transitivity: $$tsp \wedge tsq \wedge tsr \wedge tpq \wedge tqr \implies tpr$$

The same intuition also holds for the other side of the half-plane:

- Dual Transitivity:
  $$\begin{aligned} stp \wedge stq \wedge str \wedge tpq \wedge tqr \implies tpr \end{aligned}$$

Knuth shows that under the assumptions of Cyclic Symmetry, Antisymmetry, and Nondegeracy, Transitivity holds if and only if Dual Transitivity holds. Knuth requires more than half a page of low level reasoning, but as this reasoning is carried out abstractly in a small first order theory, sledgehammer (Isabelle’s interface to various automatic theorem provers) is able to find a proof that consists of just one single invocation of an automated prover.

#### Total Order from CCW

As sketched earlier, in order to compute the edges of a zonotope, we need to be able to select a “rightmost” element of a set of vectors. With the transitivity relation presented before, we can obtain a total order on vectors which allows us to do just that: Given a center *t* and another point *s*, the orientation predicate *tpq* can be used to define a total order on points *p*, *q* in the half-plane left of *ts*, i.e., $p < q$ iff *tpq*. From Antisymmetry and Nondegeneracy of the ccw system, we get antisymmetry and totality for the order <. Transitivity of the order < follows from the axiom Transitivity of the ccw system and the assumption that all points are in the half-plane left of *ts*. This ordering is then used to sort the list of generators such that they actually “wrap up” the zonotope.

#### Instantiation for Points in the Plane

Up to now, our reasoning was based abstractly on ccw systems, but of course we also want to use the results for a concrete ccw predicate. Well known from analytic geometry is the fact that ccw orientation is given by the sign of the following determinant |*pqr*|:

$$\begin{aligned} |pqr| {:}= \left| \begin{matrix} p_x &{} p_y &{} 1 \\ q_x &{} q_y &{} 1 \\ r_x &{} r_y &{} 1 \\ \end{matrix} \right| = \begin{matrix} p_x * q_y + p_y * r_x + q_x * r_y - \\ \left( r_x * q_y + r_y * p_x + q_x * p_y\right) \end{matrix} \end{aligned}$$

Points are collinear iff $|pqr| = 0$. Under the assumption that one works with a finite set of points where no three points are collinear, the following predicate $pqr^>$ satisfies the axioms of a ccw system.

$$\begin{aligned} pqr^> {:}= |pqr| > 0 \end{aligned}$$

Most axioms can easily be proved using Isabelle/HOL’s rewriting for algebraic structures. Transitivity is slightly more complicated, but can also be solved automatically after a proper instantiation of Cramer’s rule, which is easily proved automatically:

$$\begin{aligned} |tpr| = \frac{|tqr||stp| + |tpq||str|}{|stq|},\,\, \text { if } |stq| \ne 0 \end{aligned}$$

#### CCW on a Vector Space

Knuth presented his axioms with a finite set of discrete points in mind, in our case we need to talk about orientation of arbitrary points in a continuous set. We therefore require consistency of the orientation predicate when vector space operations are involved.

**Fig. 8:**
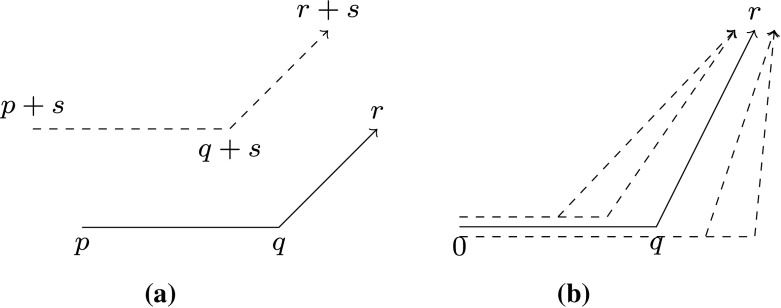
ccw axioms on a vector space. **a** Invariance under translation. **b** Invariance under scaling

One obvious requirement is that orientation is invariant under translation (Fig. 8a). With translation invariance, we can reduce every ccw triple to a triple with 0 as origin, and from there it is easy to state consistency with respect to scaling: If at *q*, there is a ccw turn to *r*, then every point on the ray from 0 through *q* will induce a ccw turn to *r* (Fig. 8b). Negative scalars can be treated by requiring that reflecting one point at the origin inverts the ccw predicate (Reflection). Furthermore, the addition of vectors *q* and *r*, which are both ccw of a line *p* needs to be ccw of *p* as well.

- Translation: $$(p + s)(q + s)(r + s)^> = pqr^>$$
- Scaling: $$\alpha> 0 \implies 0(\alpha \cdot q)r^> = 0qr^>$$
- Reflection: $$0(-p)q = 0qp$$
- Addition: $$0pq \implies 0pr \implies 0p(q + r)$$

The predicate $pqr^>$ simplifies much of the reasoning, because it satisfies the axioms of a ccw system. It does, however, ignore collinear points and therefore all the points of the zonotope that lie on its edges. In order to also include those into the reasoning, we define the slightly relaxed ccw predicate $pqr^\ge$, which holds for all points on the line through *pq* and for all points on the half-plane left of *pq*.

$$\begin{aligned} pqr^\ge {:}= |pqr|\ge 0 \end{aligned}$$

The situation is as follows: $pqr^\ge$ is the actual specification that we care about. But it does not satisfy the axioms of a ccw system, which makes reasoning very convenient. We therefore first prove the corresponding properties for the ccw system $pqr^>$. With a simple argument on continuity, the results about $pqr^>$ carry over to $pqr^\ge$ and therefore the whole zonotope.

## Program and Data Refinement

We use two different approaches to turn abstract formalizations into executable constructs: In situations where abstract operations directly correspond to concrete, executable ones, we use light-weight data refinement via the code generator. In more demanding situations, we employ a dedicated framework for nondeterministic specifications and stepwise program refinement, namely the *Autoref* tool.

### Light-Weight Data Refinement

For light-weight data refinement [10] via the code generator, abstract operations need to be mapped directly to concrete, executable ones. Examples of such abstract types are affine forms and real numbers.

Consider the type of real numbers $\mathbb {R}$. We call it abstract, because as an uncountable set, $\mathbb {R}$ is obviously not computable. But one can restrict oneself to working on a computable subset of the real numbers. In our case, we use software floating point numbers $\mathbb {F}= \{m\cdot 2^e \mid m, e \in \mathbb {Z}\}$ for (unbounded) integers $m, e \in \mathbb {Z}$. We instruct the code generator to use an uninterpreted constructor ${{\textsf {\textit{Real-of-Float}}}}\,:\mathbb {F}\rightarrow \mathbb {R}$ to represent real numbers. Operations on real numbers are then computed by pattern matching on that constructor and executing the corresponding concrete operation, e.g., for addition:

$$\begin{aligned} ({{\textsf {\textit{Real-of-Float}}}}\,~f) + ({{\textsf {\textit{Real-of-Float}}}}\,~g) = ({{\textsf {\textit{Real-of-Float}}}}\,~(f + g)) \end{aligned}$$

Since this implementing equation is proved as a theorem, such a setup does not change the trusted code base. All one has to ensure is that all abstract operations that occur in the code can be executed in the concrete representation.

Affine forms are abstractly a subtype of functions $\mathbb {N}\rightarrow \mathbb {R}$. They are implemented using a type of association lists $(\mathbb {N}\times \mathbb {R})\,{{\textsf {\textit{list}}}}\,$ that are (reverse) strictly sorted according to the keys. This sparse representation is useful because the largest index of a non-zero generator can be directly read off by inspecting only the first element. Adding a fresh generator can be done by simply prepending the new element. Binary operations are efficiently and easily implemented by merging the two lists of generators.

### Autoref

We use Lammich’s framework *Autoref* for (automatic) refinement [29, 30] in Isabelle/HOL. Autoref allows the user to specify algorithms on an abstract level and provides tool support for stepwise refinement [1] to concrete, executable implementations.

In this section we present a setup of Autoref for rigorous numerical algorithms: We provide abstract specifications for elementary operations of common rigorous numerical algorithms as well as suitable implementations.

#### Nondeterministic Specifications

An important insight when verifying algorithms that use rigorous numerical enclosures is the fact that, for correctness, any enclosure of the real quantity suffices. We model this with appropriate nondeterministic specifications.

Autoref is based on a nondeterminism monad $\alpha ~{{\textsf {\textit{nres}}}}\,$, where programs can either fail or yield a set of values as result.

$$\begin{aligned} {\mathbf {\mathsf{{datatype}}}}~\alpha ~{{\textsf {\textit{nres}}}}\,= {{\textsf {\textit{FAIL}}}}\,~|~{{\textsf {\textit{RES}}}}\,~(\alpha ~set) \end{aligned}$$

The refinement relation $\le$ on ${{\textsf {\textit{nres}}}}\,$ has ${{\textsf {\textit{FAIL}}}}\,$ as top element and ${{\textsf {\textit{RES}}}}\,~S \le {{\textsf {\textit{RES}}}}\,~T$ iff $S \subseteq T$. For deterministic results, we write ${{\textsf {\textit{return}}}}\,~x {:}= {{\textsf {\textit{RES}}}}\,~\{s\}$. We write for specifications ${{\textsf {\textit{spec}}}}\,~P {:}= {{\textsf {\textit{RES}}}}\,\{x.~P~x\}$ the result of all values satisfying the predicate *P*.

This allows one to specify correctness, e.g, of a program *f* whose inputs *x* satisfy the precondition *P* and every possible value *y* in its nondeterministic result satisfies the postcondition *Q*: $\forall x.~P~x \implies f~x \le {{\textsf {\textit{spec}}}}\,(\lambda y.~Q~y)$.

In this setting, we specify a set of operations that are useful in the context of verifying rigorous numerical algorithms, i.e., algorithms that manipulate enclosures. These operations are best modeled nondeterministically, because one is often only interested in some safe result.

Subdivisions are a means to maintain precision, we therefore have the following abstract specifications for splitting a set (with or without the possibility to perform overapproximations):

$$\begin{aligned} {{\textsf {\textit{split-spec}}}}\,_{\subseteq }~X&{:}= {{\textsf {\textit{spec}}}}\,(\lambda (A, B).~ X \subseteq A \cup B)\\ {{\textsf {\textit{split-spec}}}}\,_{=}~X&{:}= {{\textsf {\textit{spec}}}}\,(\lambda (A, B).~X = A \cup B) \end{aligned}$$

The following specifications yield some lower/upper bound on the set, not necessarily exact:

$$\begin{aligned} {{\textsf {\textit{Inf-spec}}}}\,~X&{:}= {{\textsf {\textit{spec}}}}\,(\lambda i.~\forall x \in X.~i \le x) \\ {{\textsf {\textit{Sup-spec}}}}\,~X&{:}= {{\textsf {\textit{spec}}}}\,(\lambda s.~\forall x \in X.~x \le s) \end{aligned}$$

Depending on the concrete representation of sets, one might not be able to *decide* certain properties, but only give a positive answer if the precision is sufficient. We therefore have a specification that may guarantee disjointness.

$$\begin{aligned} {{\textsf {\textit{disjoint-spec}}}}\,~X~Y {:}= {{\textsf {\textit{spec}}}}\,(\lambda b.~ b \implies X \cap Y = \emptyset ) \end{aligned}$$

As seen in the previous Sect. 4, depending on the data structure, one can not (or does not want to) compute an exact representation for the intersection of sets. These specifications allow one to overapproximate an intersection, while guaranteeing that the result does not exceed one of the arguments:

$$\begin{aligned}&{{\textsf {\textit{inter-spec}}}}\,_1~X~Y {:}= {{\textsf {\textit{spec}}}}\,(\lambda R.~X \cap Y \subseteq R \wedge R \subseteq X) \\&\quad {{\textsf {\textit{inter-spec}}}}\,_2~X~Y {:}= {{\textsf {\textit{spec}}}}\,(\lambda R.~X \cap Y \subseteq R \wedge R \subseteq Y) \end{aligned}$$

To bridge the gap to concrete numerical computations and the results from Sect. 3, we use a specification for overapproximations of evaluating straight line programs:


#### Refinement Relations

In Autoref, specifications in the ${{\textsf {\textit{nres}}}}\,$ monad are transferred to executable constructs in a structured way. Autoref is centered around a collection of so-called transfer rules. Transfer rules relate abstract with concrete operations. A transfer rule involves a transfer relation $R{:}{:}(\gamma \times \alpha )\,{{\textsf {\textit{set}}}}\,$, which relates a concrete implementation $c{:}{:}\gamma$ with an abstract element $a{:}{:}\alpha$ and is of the following form.

$$\begin{aligned} (c{:}{:}\gamma , a{:}{:}\alpha ) \in R \end{aligned}$$

Transfer rules are used to structurally synthesize concrete algorithms from abstract ones. Relations and relators (which combine relations) are used to express the relationship between concrete and abstract types.

${{\textsf {\textit{br}}}}\,$ is used to build a relation from an abstraction function $a{:}{:}\gamma \rightarrow \alpha$ and an invariant *I* on the concrete type.

$$\begin{aligned} {{\textsf {\textit{br}}}}\,~a~I {:}= \{(c,a~c) \mid I~c\} \end{aligned}$$

*Natural Relators* For the types of functions, products, sets, or data types like lists and ${{\textsf {\textit{nres}}}}\,$, one uses the natural relators $A \rightarrow _{{\textsf {\textit{r}}}}\,B, A \times _{{\textsf {\textit{r}}}}\,B, \langle A \rangle {{\textsf {\textit{set}}}}\,_{{\textsf {\textit{r}}}}\,, \langle A \rangle {{\textsf {\textit{list-rel}}}}\,, \langle A \rangle {{\textsf {\textit{nres}}}}\,_{{\textsf {\textit{r}}}}\,$ with relations *A*, *B* for the argument types.

$$\begin{aligned} (f, f') \in A \rightarrow _{{\textsf {\textit{r}}}}\,B&\iff \forall (x, y) \in A.~(f~x, f'~x) \in B \\ ((a, b), (a', b')) \in A \times _{{\textsf {\textit{r}}}}\,B&\iff (a, a') \in A \wedge (b, b') \in B \\ (X, X') \in \langle A \rangle {{\textsf {\textit{set}}}}\,_{{\textsf {\textit{r}}}}\,&\iff (\forall x\in X.~\exists x' \in X'.~(x, x') \in A)~\wedge \\&\phantom {\iff ~~} (\forall x'\in X'.~\exists x \in X.~(x, x') \in A) \\ (xs, xs') \in \langle A \rangle {{\textsf {\textit{list-rel}}}}\,&\iff {{\textsf {\textit{length}}}}\,~xs = {{\textsf {\textit{length}}}}\,~xs' ~\wedge \\&\phantom {\iff ~~} (\forall i<{{\textsf {\textit{length}}}}\,~xs.~(xs_i, xs'_i) \in A) \\ ({{\textsf {\textit{RES}}}}\,~X, {{\textsf {\textit{RES}}}}\,~X') \in \langle A \rangle {{\textsf {\textit{nres}}}}\,_{{\textsf {\textit{r}}}}\,&\iff (X, X') \in \langle A \rangle {{\textsf {\textit{set}}}}\,_{{\textsf {\textit{r}}}}\,\end{aligned}$$

*Representing Vectors* We represent vectors (an arbitrary type $\alpha$ of class Euclidean space) as lists of real numbers where the length matches the dimension of the Euclidean space.

$$\begin{aligned} {{\textsf {\textit{lv-rel}}}}\,{:}= {{\textsf {\textit{br}}}}\,~{{\textsf {\textit{eucl-of-list}}}}\,~(\lambda xs.~{{\textsf {\textit{len}}}}\,~xs = DIM(\alpha )) \end{aligned}$$

This way, the concrete algorithm is monomorphic, which has the advantage that it can be generated once and for all and can therefore be used as a stand-alone tool.

*Representing Enclosures* We provide several implementations for the sets that can be used as enclosures. Intervals are represented by pairs of element types (which, in turn are implemented via some relation *A*):

$$\begin{aligned} \langle A \rangle {{\textsf {\textit{ivl}}}}\,_{{\textsf {\textit{r}}}}\,{:}= \{((a', b'), [a , b]) \mid (a', a) \in A \wedge (b', b) \in A \} \end{aligned}$$

Zonotopes are represented using the joint range ${{\textsf {\textit{joint-range}}}}\,$ of affine forms

$$\begin{aligned} {{\textsf {\textit{affine}}}}\,_{{\textsf {\textit{r}}}}\,{:}= {{\textsf {\textit{br}}}}\,~(\lambda A.~{{\textsf {\textit{eucl-of-list}}}}\,({{\textsf {\textit{joint-range}}}}\,~A))~(\lambda \_.~{{\textsf {\textit{True}}}}\,) \end{aligned}$$

We use a symbolic representation of planes using the data type constructor ${{\textsf {\textit{Sctn}}}}\,$ that keeps normal vector *n* and translation *c* of a hyperplane. It is interpreted using

$$\begin{aligned}&{{\textsf {\textit{plane-of}}}}\,({{\textsf {\textit{Sctn}}}}\,~n~c) {:}= \{ x \mid \langle x, n\rangle = c \} \\&\quad {{\textsf {\textit{halfspace}}}}\,({{\textsf {\textit{Sctn}}}}\,~n~c) {:}= \{ x \mid \langle x, n\rangle \le c\} \end{aligned}$$

for the hyperplane itself or for the halfspace below the hyperplane, where $\langle x, n\rangle$ is the inner product (also called dot product). $\langle A \rangle {{\textsf {\textit{sctn}}}}\,_{{\textsf {\textit{r}}}}\,$ is the natural relator that allows one to change the representation of the normal vector. With this, we can give a concrete implementation for hyperplanes and half-spaces.

$$\begin{aligned}&\langle A \rangle {{\textsf {\textit{plane}}}}\,_{{\textsf {\textit{r}}}}\,{:}= \langle A\rangle {{\textsf {\textit{sctn}}}}\,_{{\textsf {\textit{r}}}}\,\circ {{\textsf {\textit{br}}}}\,~{{\textsf {\textit{plane-of}}}}\,~(\lambda \_.~{{\textsf {\textit{True}}}}\,) \\&\quad \langle A \rangle {{\textsf {\textit{halfspace}}}}\,_{{\textsf {\textit{r}}}}\,{:}= \langle A\rangle {{\textsf {\textit{sctn}}}}\,_{{\textsf {\textit{r}}}}\,\circ {{\textsf {\textit{br}}}}\,~{{\textsf {\textit{halfspace}}}}\,~(\lambda \_.~{{\textsf {\textit{True}}}}\,) \end{aligned}$$

For those relations, ${{\textsf {\textit{plane-of}}}}\,$, ${{\textsf {\textit{halfspace}}}}\,$, and $\cap$ are easily implemented with the identity function or as pair. On the abstract level, they describe useful objects that are convenient to reason about.

$$\begin{aligned} ((\lambda x.~x), {{\textsf {\textit{plane-of}}}}\,) \in \langle A \rangle {{\textsf {\textit{sctn}}}}\,_{{\textsf {\textit{r}}}}\,\rightarrow _{{\textsf {\textit{r}}}}\,\langle A \rangle {{\textsf {\textit{plane}}}}\,_{{\textsf {\textit{r}}}}\,\\ ((\lambda x.~x), {{\textsf {\textit{halfspace}}}}\,) \in \langle A \rangle {{\textsf {\textit{sctn}}}}\,_{{\textsf {\textit{r}}}}\,\rightarrow _{{\textsf {\textit{r}}}}\,\langle A \rangle {{\textsf {\textit{halfspace}}}}\,_{{\textsf {\textit{r}}}}\,\\ ((\lambda x~y.~(x, y)), \cap ) \in A \rightarrow _{{\textsf {\textit{r}}}}\,B \rightarrow _{{\textsf {\textit{r}}}}\,\langle A, B\rangle {{\textsf {\textit{inter}}}}\,_{{\textsf {\textit{r}}}}\,\end{aligned}$$

We will see that in some algorithms, one maintains a collection of enclosures, but abstractly one likes to see them as just one enclosure. For a relation $A:(\beta \times \alpha \,{{\textsf {\textit{set}}}}\,)\,{{\textsf {\textit{set}}}}\,$ that implements single enclosures for sets of type $\alpha$ with some concrete representation of type $\beta$, and a relation $S:(\sigma \times \beta \,{{\textsf {\textit{set}}}}\,)\,{{\textsf {\textit{set}}}}\,$ that implements sets of concrete elements $\beta$, we define a relation that represents the union of all those elements as follows:

$$\begin{aligned}&\langle S, A\rangle {{\textsf {\textit{Union}}}}\,_{{\textsf {\textit{r}}}}\,: (\sigma \times \alpha \,{{\textsf {\textit{set}}}}\,)\,{{\textsf {\textit{set}}}}\, \\&\quad \langle S, A\rangle {{\textsf {\textit{Union}}}}\,_{{\textsf {\textit{r}}}}\,{:}= S \circ \langle A \rangle {{\textsf {\textit{set}}}}\,_{{\textsf {\textit{r}}}}\,\circ {{\textsf {\textit{br}}}}\,~\left( \lambda X. \bigcup _{x\in X} x\right) (\lambda \_.~{{\textsf {\textit{True}}}}\,) \end{aligned}$$

Currently, we use lists to implement the set of concrete representations *S*, for which we write $\langle A\rangle {{\textsf {\textit{Union}}}}\,_{{{\textsf {\textit{lr}}}}\,}{:}= \langle {{\textsf {\textit{list-set}}}}\,_{{\textsf {\textit{r}}}}\,, A \rangle {{\textsf {\textit{Union}}}}\,_{{\textsf {\textit{r}}}}\,$, and operations like union or extracting one element (with the specification ${{\textsf {\textit{split-spec}}}}\,_{=}$) can be implemented with the respective operations on lists/sets:

$$\begin{aligned} (\lambda xs~ys.~{{\textsf {\textit{return}}}}\,(xs @ ys), \cup )&\in \langle A \rangle {{\textsf {\textit{Union}}}}\,_{{{\textsf {\textit{lr}}}}\,}\rightarrow _{{\textsf {\textit{r}}}}\,\langle A \rangle {{\textsf {\textit{Union}}}}\,_{{{\textsf {\textit{lr}}}}\,}\rightarrow _{{\textsf {\textit{r}}}}\,\langle A \rangle {{\textsf {\textit{Union}}}}\,_{{{\textsf {\textit{lr}}}}\,}\\ (\lambda x.~{{\textsf {\textit{return}}}}\,({{\textsf {\textit{hd}}}}\,x, {{\textsf {\textit{tl}}}}\,~x), {{\textsf {\textit{split-spec}}}}\,_{=})&\in \langle A \rangle {{\textsf {\textit{Union}}}}\,_{{{\textsf {\textit{lr}}}}\,}\rightarrow _{{\textsf {\textit{r}}}}\,\langle A \times _{{\textsf {\textit{r}}}}\,\langle A \rangle {{\textsf {\textit{Union}}}}\,_{{{\textsf {\textit{lr}}}}\,}\rangle {{\textsf {\textit{nres}}}}\,_{{\textsf {\textit{r}}}}\,\end{aligned}$$

*Relations to Guide Heuristics* Often, in particular to guide heuristics, an algorithm needs to carry around information, which does not influence correctness proofs. An ODE solver for example, modifies its step size, also based on previous values. An implementation needs to carry this information around, but for verifying the algorithm, this only introduces unnecessary clutter. We therefore introduce a relation that carries more information (implemented via *A*) in the implementation, but keeps the abstract semantics (implemented via *B*):

$$\begin{aligned} \langle A, B\rangle {{\textsf {\textit{info}}}}\,_{{\textsf {\textit{r}}}}\,{:}= \{((a', b'), b) \mid \exists a.~(a', a) \in A \wedge (b', b) \in B\} \end{aligned}$$

Adding information is simply done by using a pair in the implementation side. Semantically, this information is simply discarded (${{\textsf {\textit{put-info}}}}\,~a~b {:}= b$). Information can be extracted with ${{\textsf {\textit{get-info}}}}\,$, which is semantically just an arbitrary element (${{\textsf {\textit{get-info}}}}\,~b {:}= {{\textsf {\textit{spec}}}}\,(\lambda \_.~{{\textsf {\textit{True}}}}\,)$). The implementations are straightforward:

$$\begin{aligned} (\lambda a~b.~(a, b), {{\textsf {\textit{put-info}}}}\,)&\in A \rightarrow _{{\textsf {\textit{r}}}}\,B \rightarrow _{{\textsf {\textit{r}}}}\,\langle A, B\rangle {{\textsf {\textit{info}}}}\,_{{\textsf {\textit{r}}}}\,\\ ((\lambda (a, b). {{\textsf {\textit{return}}}}\,~a), {{\textsf {\textit{get-info}}}}\,)&\in \langle A, B\rangle {{\textsf {\textit{info}}}}\,_{{\textsf {\textit{r}}}}\,\rightarrow \langle A \rangle {{\textsf {\textit{nres}}}}\,_{{\textsf {\textit{r}}}}\,\end{aligned}$$

An example of its usage is illustrated later on in Algorithm 1.

## Reachability Analysis

**Fig. 9:**
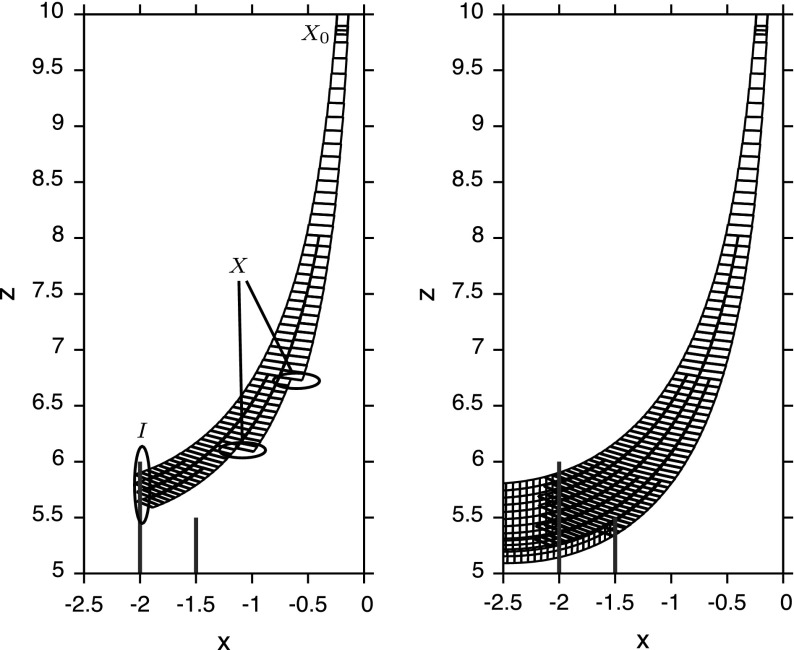
Continuous reachability and intermediate Poincaré sections

Overall, we design an algorithm that computes a Poincaré map with a list of intermediate Poincaré sections. The global idea (illustrated in Fig. 9) is as follows: starting from a set $X_0$, perform a series of single time discretization steps. If reachable sets grow above a given threshold, subdivide them (Sects. 6.3 and 6.4). Stop before an intermediate (or the final) section would be hit, then resolve the Poincaré map at that section (Sect. 6.5). For Tucker’s proof, it is important to also track the matrix of partial derivatives together with the solution. To this end, one can encode the derivative as a higher-dimensional ODE and use essentially the same algorithms as before. This instrumentation is presented in Sect. 6.7.

### The Framework

We use the high-level constructors and abstract specifications from the previous Sect. 5. We remain agnostic about the type of enclosures, for which we assume a relation ${{\textsf {\textit{encl}}}}\,_{{\textsf {\textit{r}}}}\,$ and implementations for the abstract operations that are needed for the reachability analysis algorithms: an approximation scheme for expressions ${{\textsf {\textit{approx-slp-spec}}}}\,$, enclosures from intervals using an implementation ${{\textsf {\textit{encl-of-ivl}}}}\,$, lower and upper bounds with ${{\textsf {\textit{Inf-spec}}}}\,, {{\textsf {\textit{Sup-spec}}}}\,$, intersections with a plane ${{\textsf {\textit{inter-spec}}}}\,_2$ (note that the relation fixes the second argument to represent a plane, abstractly ${{\textsf {\textit{inter-spec}}}}\,_2$ is just intersection on sets):

- $$({{\textsf {\textit{approx-slp}}}}\,, {{\textsf {\textit{approx-slp-spec}}}}\,) \in {{\textsf {\textit{slp}}}}\,_{{\textsf {\textit{r}}}}\,\rightarrow _{{\textsf {\textit{r}}}}\,{{\textsf {\textit{encl}}}}\,_{{\textsf {\textit{r}}}}\,\rightarrow _{{\textsf {\textit{r}}}}\,\langle \langle {{\textsf {\textit{encl}}}}\,_{{\textsf {\textit{r}}}}\,\rangle {{\textsf {\textit{option}}}}\,_{{\textsf {\textit{r}}}}\,\rangle {{\textsf {\textit{nres}}}}\,_{{\textsf {\textit{r}}}}\,$$
- $$(\lambda x~y.~{{\textsf {\textit{encl-of-ivl}}}}\,~x~y, \lambda x~y.~[x, y]) \in {{\textsf {\textit{lv-rel}}}}\,\rightarrow _{{\textsf {\textit{r}}}}\,{{\textsf {\textit{lv-rel}}}}\,\rightarrow _{{\textsf {\textit{r}}}}\,{{\textsf {\textit{encl}}}}\,_{{\textsf {\textit{r}}}}\,$$
- $$({{\textsf {\textit{inf-encl}}}}\,, {{\textsf {\textit{Inf-spec}}}}\,) \in {{\textsf {\textit{encl}}}}\,_{{\textsf {\textit{r}}}}\,\rightarrow _{{\textsf {\textit{r}}}}\,\langle {{\textsf {\textit{lv-rel}}}}\,\rangle {{\textsf {\textit{nres}}}}\,_{{\textsf {\textit{r}}}}\,$$
- $$({{\textsf {\textit{sup-encl}}}}\,, {{\textsf {\textit{Sup-spec}}}}\,) \in {{\textsf {\textit{encl}}}}\,_{{\textsf {\textit{r}}}}\,\rightarrow _{{\textsf {\textit{r}}}}\,\langle {{\textsf {\textit{lv-rel}}}}\,\rangle {{\textsf {\textit{nres}}}}\,_{{\textsf {\textit{r}}}}\,$$
- $$({{\textsf {\textit{split-encl}}}}\,, {{\textsf {\textit{split-spec}}}}\,_{\subseteq }) \in {{\textsf {\textit{real}}}}\,_{{\textsf {\textit{r}}}}\,\rightarrow _{{\textsf {\textit{r}}}}\,nat_rel \rightarrow _{{\textsf {\textit{r}}}}\,{{\textsf {\textit{encl}}}}\,_{{\textsf {\textit{r}}}}\,\rightarrow _{{\textsf {\textit{r}}}}\,\langle {{\textsf {\textit{encl}}}}\,_{{\textsf {\textit{r}}}}\,\times _{{\textsf {\textit{r}}}}\,{{\textsf {\textit{encl}}}}\,_{{\textsf {\textit{r}}}}\,\rangle {{\textsf {\textit{nres}}}}\,_{{\textsf {\textit{r}}}}\,$$
- $$({{\textsf {\textit{inter-encl-plane}}}}\,, {{\textsf {\textit{inter-spec}}}}\,_2) \in {{\textsf {\textit{encl}}}}\,_{{\textsf {\textit{r}}}}\,\rightarrow _{{\textsf {\textit{r}}}}\,\langle {{\textsf {\textit{lv-rel}}}}\,\rangle {{\textsf {\textit{plane}}}}\,_{{\textsf {\textit{r}}}}\,\rightarrow _{{\textsf {\textit{r}}}}\,\langle {{\textsf {\textit{encl}}}}\,_{{\textsf {\textit{r}}}}\,\rangle {{\textsf {\textit{nres}}}}\,_{{\textsf {\textit{r}}}}\,$$

Currently, the only instantiation of this scheme is with affine arithmetic (in this case we set ${{\textsf {\textit{encl}}}}\,_{{\textsf {\textit{r}}}}\,$ to ${{\textsf {\textit{affine}}}}\,_{{\textsf {\textit{r}}}}\,$). Nevertheless, this structure keeps the formalization modular and one can imagine to add further instantiations—with e.g., Taylor models or centered forms—in the future.

### The Specification

Our algorithms are supposed to compute enclosures for solutions of the ODE. We formalize the enclosure of an evolution from an initial set *X* to some other set *Y* with the ternary predicate $\curvearrowright$, where $X \curvearrowright _{C} Y$ holds if the evolution flows every point of $X \subseteq \mathbb {R}^{n}$ to some point in $Y\subseteq \mathbb {R}^{n}$ and does not leave the set *C* in the meantime. We call *C* the *flowpipe* from *X* to *Y*.

#### Definition 9

(*Flows-to Predicate*)

$$\begin{aligned} X \curvearrowright _{C} Y {:}= \forall x \in X.~\exists t\ge 0.~\phi (x_0, t) \in Y \wedge (\forall 0 \le s \le t.~\phi (x_0, s) \in C_X) \end{aligned}$$

### Single Step

In order to compute enclosures for a single step, one needs to first certify that a solution exists, which is the case for an initial value $x_0$ and stepsize *h* if the iteration given by the Picard iteration from Sect. 2.1.2 has a unique fixed point. This is the standard approach from Bouissou et al. [4], also described in the setting of Isabelle [18]. The idea is that the expression $Q_h(X) = X_0 + [0,h]\cdot f(X)$, which we can evaluate using ${{\textsf {\textit{approx-slp-spec}}}}\,$, is an overapproximation of the Picard iteration and a post-fixed point certifies existence and a crude enclosure for solutions up to time *h*. This crude enclosure can be used as an overapproximation for the terms $x (hs_1 + t)$ in the Runge–Kutta approximation scheme from Sect. 3.2. The function ${{\textsf {\textit{rk-step}}}}\,$ implements this and actually evaluates the Runge–Kutta approximation scheme twice: once for time *h* and once for the time interval [0, *h*], because this gives a much better enclosure for the flowpipe up to time *h* than the crude overapproximation from the Picard iteration. We prove the following specification.

#### Theorem 10

$${{\textsf {\textit{rk-step}}}}\,~X~h \le {{\textsf {\textit{spec}}}}\,(\lambda (\varepsilon , C, Y).~X \curvearrowright _C Y)$$

The returned value $\epsilon$ is an estimate for the approximation error. This is used for an adaptive step size control. Algorithm 1 shows an example how to use this heuristic (and another heuristic to split large sets), while (almost trivially, because the additional operations are either vacuous, the identity or overapproximations) satisfying the same specification.

#### Theorem 11

$${{\textsf {\textit{single-step}}}}\,~X~h \le {{\textsf {\textit{spec}}}}\,(\lambda (C, Y). X \curvearrowright _C Y)$$

The information on the last (and next) step size is only reflected in the refinement relation for the implementation of *single-step* :

$$\begin{aligned}&({{\textsf {\textit{single-step}}}}\,_{{\textsf {\textit{impl}}}}\,, {{\textsf {\textit{single-step}}}}\,) \in \\&\qquad \langle {{\textsf {\textit{real}}}}\,_{{\textsf {\textit{r}}}}\,, {{\textsf {\textit{encl}}}}\,_{{\textsf {\textit{r}}}}\,\rangle {{\textsf {\textit{info}}}}\,_{{\textsf {\textit{r}}}}\,\rightarrow \langle \langle \langle {{\textsf {\textit{real}}}}\,_{{\textsf {\textit{r}}}}\,, {{\textsf {\textit{encl}}}}\,_{{\textsf {\textit{r}}}}\,\rangle {{\textsf {\textit{info}}}}\,_{{\textsf {\textit{r}}}}\,\rangle {{\textsf {\textit{Union}}}}\,_{{{\textsf {\textit{lr}}}}\,}, \langle {{\textsf {\textit{encl}}}}\,_{{\textsf {\textit{r}}}}\,\rangle {{\textsf {\textit{Union}}}}\,_{{{\textsf {\textit{lr}}}}\,}\rangle {{\textsf {\textit{nres}}}}\,_{{\textsf {\textit{r}}}}\,\end{aligned}$$

But this information does not clutter the verification of ${{\textsf {\textit{single-step}}}}\,$ or the statement of Theorem 11, which is very convenient.
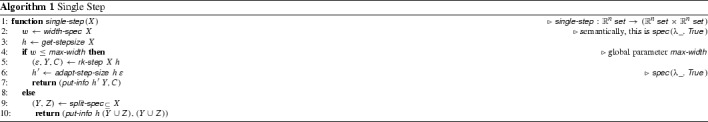


### Continuous Reachability


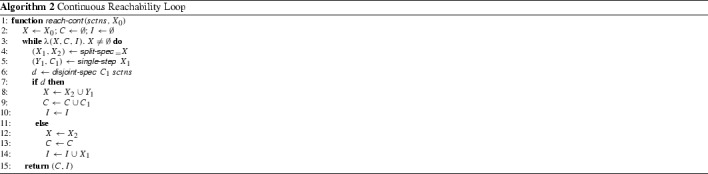


Note that ${{\textsf {\textit{single-step}}}}\,$ returns (from an implementation point of view) a collection of enclosures, so we need some sort of work-list algorithm to resolve all currently reachable sets. Algorithm 2 does so. It maintains three kinds of sets (see also Fig. 9): *X* is the collection of currently “live” sets. *C* is the collection of all flowpipes explored so far. *I* is the collection of sets where reachability analysis has stopped because of an intersection with a Poincaré section from *sctns*. The algorithm takes one element out of the “work-list” *X* by splitting the collection of enclosures using ${{\textsf {\textit{split-spec}}}}\,_{=}$, performs a single step, checks for an intersection with one of the Poincaré sections and updates *X*, *C*,  and *I* accordingly.

The loop invariant of ${{\textsf {\textit{reach-cont}}}}\,$ is roughly the following: Elements from $X_0$ flow via *C* to *X* and *I*, while avoiding *sctns*.

$$\begin{aligned} X_0 \curvearrowright _{C} (X \cup I) \wedge C \cap sctns = \emptyset \end{aligned}$$

The specification of ${{\textsf {\textit{reach-cont}}}}\,$ follows immediately:

#### Theorem 12

$${{\textsf {\textit{reach-cont}}}}\,(sctns, X_0) \le {{\textsf {\textit{spec}}}}\,(\lambda (C, I).~X_0 \curvearrowright _{C} I \wedge C \cap sctns = \emptyset )$$

It is worth noticing that the simplicity of the statement of this correctness theorem is due to the fact that the work-list and heuristic info is hidden via refinement relations in the implementation. If this were represented on the specification level (e.g., by using sets of enclosures paired with their current step size), the specification would have to be much more cluttered:

$$\begin{aligned} \left( \bigcup _{(h, x)\in X_0} x\right) \curvearrowright _{C} \left( \left( \bigcup _{(h, x)\in X} x\right) \cup \left( \bigcup I\right) \right) \end{aligned}$$

Such a specification distracts the user and also automatic proof tools—we are therefore happy to hide this in the abstraction.

### Resolve Intersection

Algorithm 2 performs reachability analysis until each enclosure is just about to intersect an hyperplane. We compute the intersection from an enclosure *X* with a hyperplane *H* again in an iteration: continuous reachability steps are repeated as long as flowpipes intersect the hyperplane. The intersection of the individual flowpipes is computed with the geometric algorithm from Sect. 4.

When the intersection is computed by flowing the reachable set through the hyperplane step by step, we get a set $\mathcal {I}$ consisting of individual intersections $I_i$. Many of the sets $I_i$ usually overlap, in order to avoid redundant enclosures, the overlap is resolved with an overapproximative operation: all of the sets $I_i$ are covered with an interval, which is repeatedly subdivided and shrinked to a given precision (see also section 3.6 in [21] for a more detailed discussion).

### Intermediate Poincaré Maps

The overall algorithm repeats the alternation of continuous reachability and resolution of Poincaré sections. When there is a sequence $H_1, \cdots , H_n$ of intermediate Poincaré maps to be computed, it is important to ensure that, while flowing towards or resolving section $H_i$, one must not intersect with any of the $H_j$ with $j>i$. Otherwise the later computation of the Poincaré map $H_j$ might be incorrect because the actual first return time was reached before.

### Derivatives

For Tucker’s proof, it is necessary to compute not only the Poincaré map, but also its derivative. The derivative of the flow can be encoded as a higher dimensional ODE according to the variational equation (Theorem 7).

For an ODE with right hand side $f:\mathbb {R}^{n}\rightarrow \mathbb {R}^{n}$, a new ODE of type $\mathbb {R}^{n} \times \mathbb {R}^{n*n}$ with right hand side $(x, W) \mapsto (f~x, \mathsf {D}f|_{x} \cdot W)$ is constructed. Here the first component contains the solution, and the second component its matrix of derivatives.

We first extend the flows-to predicate $\curvearrowright$ to a predicate $\curvearrowright '$ which also takes derivatives into account.

#### Definition 10

(*Flows-to Predicate Extended with Derivative*)

$$\begin{aligned} X \curvearrowright '_{C} Y&{:}= \forall (x, d) \in X.~\exists t\ge 0.~(\phi (x_0, t), \mathsf {D}\phi |_{(x_0, t)} \cdot _{{{\textsf {\textit{bl}}}}\,}d) \in Y~\wedge \\&\qquad (\forall 0 \le s \le t.~(\phi (x_0, s), \mathsf {D}\phi |_{(x_0, s)} \cdot _{{{\textsf {\textit{bl}}}}\,}d) \in C_X) \end{aligned}$$

With this extended predicate for reachability, we can show that ${{\textsf {\textit{reach-cont}}}}\,'$, i.e., ${{\textsf {\textit{reach-cont}}}}\,$ for the extended ODE satisfies the specification ${{\textsf {\textit{reach-cont}}}}\,'~sctns~X_0' \le (\lambda (C, Y).~X \curvearrowright '_{C} Y )$.

The Poincaré map, however requires extra care, because we cannot simply intersect the derivative of the flow with the Poincaré section: the derivative of the Poincaré map is given according to the expression in Theorem 8. For a hyperplane $H = \{x \mid \langle x, n\rangle = c\}$, the derivative is given as follows (for $x \in \{x \mid \langle x, n\rangle = c\})$:

$$\begin{aligned} \mathsf {D}P|_{\varphi (t)}\cdot d = \mathsf {D}\phi |_{(x, \tau (x))}\cdot d - \frac{\langle \mathsf {D}\phi |_{(x, \tau (x))}\cdot d, n\rangle }{\langle f(P(x)), n\rangle }f(P(x)) \end{aligned}$$

We can evaluate this expression using affine arithmetic. But we need to be able to enclose all quantities that occur on the right hand side, in particular $P(x) = \varphi (x, \tau (x))$ and $\mathsf {D}\phi |_{(x, \tau (x))}\cdot d$. But we can enclose those: assume a step in computing an intersection, i.e., $X \curvearrowright _{C} Y$. Let us assume for simplicity that $(X \cup Y)\cap H = \emptyset$ and *X* and *Y* are on opposite sides of the hyperplane. Then the intersection of the flowpipe *C* with the section *H* encloses the Poincaré map: $P(X) = \{\varphi (x, \tau (x)) \mid x \in X\} \subseteq C \cap H$. For an extended flow $X' \curvearrowright _{C'} Y'$, this means $\{(\phi (x, \tau (x)), \mathsf {D}\phi |_{(x, \tau (x))}\cdot _{{{\textsf {\textit{bl}}}}\,}d) \mid (x, d) \in X'\} \subseteq C' \cap H \times \mathbb {R}^{n*n}$. Therefore both $P(x) = \phi (x, \tau (x))$ and $\mathsf {D}\phi |_{(x, \tau (x))}\cdot d$ are enclosed by the result of the intersection $C' \cap H \times \mathbb {R}^{n*n}$ for which we can use the regular intersection algorithm from Sect. 4.

### Correctness Theorem

We call the main algorithm that we outlined in the beginning of this Sect. 6 ${{\textsf {\textit{poincare}}}}\,$: It resolves a sequence of intermediate Poincaré maps (together with their derivative). It is verified to compute guaranteed enclosures for Poincaré maps and their derivative. The algorithm ${{\textsf {\textit{poincare}}}}\,$ takes as arguments an initial set $X:\mathbb {R}^{n}\,{{\textsf {\textit{set}}}}\,$, and initial matrix of partial derivatives $DX:\mathbb {R}^{n*n}\,{{\textsf {\textit{set}}}}\,$ and a target Poincaré section ${\varSigma }:\mathbb {R}^{n}\,{{\textsf {\textit{set}}}}\,$. It is further parameterized by a list of intermediate Poincaré sections, but they are irrelevant for the final correctness theorem. We formally verify partial correctness: If the algorithm returns a result, then this result encloses the Poincaré map *P*(*x*) and its derivative $\mathsf {D}P|_{x} \cdot DX$ for every $x \in X$ and $DX:\mathbb {R}^{n*n}\,{{\textsf {\textit{set}}}}\,$.

#### Theorem 13

(Correctness of ODE solver with Poincaré maps)

$$\begin{aligned} {{\textsf {\textit{poincare}}}}\,~X~DX~{\varSigma }= R \implies \forall x\in X.~(P(x), \mathsf {D}P|_{x}\cdot DX) \in R \end{aligned}$$

## Application to Lorenz Attractor

In this section, we present how the verified algorithm ${{\textsf {\textit{poincare}}}}\,$ of the previous section is used to certify Tucker’s computations. We show in particular how we formally prove the Theorems 1 and 2. It helps to recall the roles of the forward invariant set *N*, the cone field $\mathfrak {C}$ and the expansion estimates $\mathcal {E}$ in Tucker’s proof, as outlined in Sect. 1.3.

### The Input Data and its Interpretation

It is not necessary to verify precisely the set *N* that Tucker used, but coming up with a forward invariant set is slightly more involved than certifying one. We therefore use the output of Tucker’s program as a starting point to set up the input for our ODE solver. Since any other forward invariant with suitable cone field and expansion estimates would do just, we are free to modify Tucker’s data slightly. The output of Tucker’s program is available online^3^ as a file containing 7258 lines. We preprocessed this file by merging the information of some of the lines and slightly coarsening some of the numerical bounds. The coarsening accounts for slight differences between Tucker’s and our approximations.

This results in a list of 400 elements, which we call ${{\textsf {\textit{input-data}}}}\,$ and will be the basis for all further interpretations:

#### Definition 11

(*Input Data*) ${{\textsf {\textit{input-data}}}}\,{:}{:} {{\textsf {\textit{result}}}}\,\,{{\textsf {\textit{list}}}}\,$ is a list of 400 elements of type ${{\textsf {\textit{result}}}}\,$.

$$\begin{aligned} {\mathbf {\mathsf{{datatype}}}}~{{\textsf {\textit{result}}}}\,&= {{\textsf {\textit{Result}}}}\,~({{\textsf {\textit{invoke-nf}}}}\,: \mathbb {B}) \\&({{\textsf {\textit{angle}}}}\,^{-}: \mathbb {R}) ~ ({{\textsf {\textit{angle}}}}\,^{+}: \mathbb {R}) \\&({{\textsf {\textit{expansion}}}}\,: \mathbb {R}) ~ ({{\textsf {\textit{preexpansion}}}}\,: \mathbb {R}) \\&({{\textsf {\textit{gridx}}}}\,^{-}: \mathbb {Z})~({{\textsf {\textit{gridx}}}}\,^{+}: \mathbb {Z}) ~({{\textsf {\textit{gridy}}}}\,^{-}: \mathbb {Z})~({{\textsf {\textit{gridy}}}}\,^{+}: \mathbb {Z}) \\&({{\textsf {\textit{retx}}}}\,^{-}: \mathbb {Z})~({{\textsf {\textit{rety}}}}\,^{-}: \mathbb {Z})~({{\textsf {\textit{retx}}}}\,^{+}: \mathbb {Z})~({{\textsf {\textit{rety}}}}\,^{+}: \mathbb {Z}) \end{aligned}$$

**Fig. 10:**
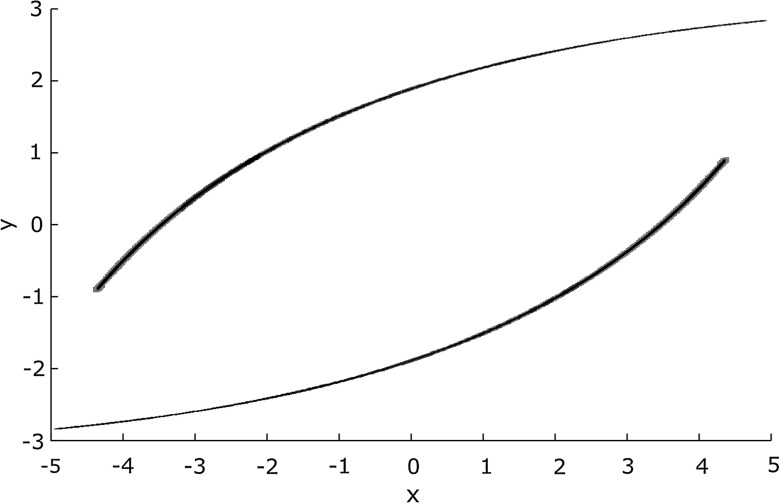
*N* in gray ($N^+$ the upper and $N^- = S(N^+)$ the lower branch) and enclosure of $P(N^+)$ in black. This is a subset of the Poincaré section ${\varSigma }$ (as in Fig. 2)

Elements *res* of type ${{\textsf {\textit{result}}}}\,$ are interpreted as initial rectangles as follows. The properties ${{\textsf {\textit{gridx}}}}\,^{-}, {{\textsf {\textit{gridx}}}}\,^{+}, {{\textsf {\textit{gridy}}}}\,^{-}$, and ${{\textsf {\textit{gridy}}}}\,^{+}$ encode a rectangle on the Poincaré section ${\varSigma }$ (recall Fig. 2), which we denote by *N*(*res*). The union of all elements of ${{\textsf {\textit{input-data}}}}\,$ represents the upper branch $N^+$ of the forward invariant set *N*. It is plotted in Fig. 10.

#### Definition 12

$$\begin{aligned} N(res) {:}=&[(({{\textsf {\textit{gridx}}}}\,^{-}~res - 1)\cdot 2^{-8}, ({{\textsf {\textit{gridy}}}}\,^{-}~res - 1)\cdot 2^{-8}, 27),\\&(({{\textsf {\textit{gridx}}}}\,^{+}~res + 1)\cdot 2^{-8}, ({{\textsf {\textit{gridy}}}}\,^{+}~res + 1)\cdot 2^{-8}, 27)] \\ N^+&{:}= \bigcup _{res\in {{\textsf {\textit{input-data}}}}\,} N(res) \\ N^-&{:}= \{(-x, -y, z) \mid (x, y, z) \in (N^+)\} \\ N&{:}= N^+ \cup N^- \\ \end{aligned}$$

The input data also contains information on the image of an initial rectangle. It is encoded in ${{\textsf {\textit{retx}}}}\,^{-}, {{\textsf {\textit{rety}}}}\,^{-}, {{\textsf {\textit{retx}}}}\,^{+}, {{\textsf {\textit{rety}}}}\,^{+}$: We select the elements within those bounds with ${{\textsf {\textit{return-of}}}}\,$:

$$\begin{aligned}&{{\textsf {\textit{return-of}}}}\,~res {:}= \{res' \in {{\textsf {\textit{input-data}}}}\,\mid \\&\qquad {{\textsf {\textit{gridx}}}}\,^{-}~res' \in [{{\textsf {\textit{retx}}}}\,^{-}~res, {{\textsf {\textit{retx}}}}\,^{+}~res]~\wedge \\&\qquad {{\textsf {\textit{gridy}}}}\,^{-}~res' \in [{{\textsf {\textit{rety}}}}\,^{-}~res, {{\textsf {\textit{rety}}}}\,^{+}~res]\} \end{aligned}$$

${{\textsf {\textit{angle}}}}\,^{-}$ and ${{\textsf {\textit{angle}}}}\,^{+}$ define the cone $\mathfrak {C}$ associated with the rectangle: the conic hull of the line segment between the boundary vectors.

#### Definition 13

$$\begin{aligned} \mathfrak {C}~res =&{{\textsf {\textit{cone}}}}\,~{{\textsf {\textit{hull}}}}\,~({{\textsf {\textit{segment}}}}\,\\&\qquad (\cos ({{\textsf {\textit{rad}}}}\,~({{\textsf {\textit{angle}}}}\,^{-}~res), \sin ({{\textsf {\textit{rad}}}}\,~({{\textsf {\textit{angle}}}}\,^{-}~res), 0)\\&\qquad (\cos ({{\textsf {\textit{rad}}}}\,~({{\textsf {\textit{angle}}}}\,^{+}~res)),\sin ({{\textsf {\textit{rad}}}}\,~({{\textsf {\textit{angle}}}}\,^{+}~res), 0))))) \end{aligned}$$

There ${{\textsf {\textit{rad}}}}\,~x = \frac{x\cdot \pi }{180}$ is the radian of the angle given in degrees, ${{\textsf {\textit{segment}}}}\,~x~y$ is the line segment $\{(1 - u) \cdot a + u \cdot b \mid u \in [0, 1]\}$, and ${{\textsf {\textit{cone}}}}\,~{{\textsf {\textit{hull}}}}\,~S = \{c \cdot x \mid 0 \le c \wedge x \in S\}$ the conic hull of a set *S*.

The elements in ${{\textsf {\textit{input-data}}}}\,$ also encode a conefield $\mathfrak {C}$ and expansion estimates as follows. ${{\textsf {\textit{results-at}}}}\,(x)$ yields the set of result elements that cover a point *x* (the rectangles overlap at the boundary). We need to respect this to ensure that $\mathfrak {C}$, $\mathcal {E}$, and $\mathcal {E}^{-1}$ are well defined.

#### Definition 14

$$\begin{aligned}&{{\textsf {\textit{results-at}}}}\,(x) {:}= \{res \in {{\textsf {\textit{input-data}}}}\,\mid x \in N(res)\} \\&\mathfrak {C}(x) {:}= \bigcup _{res \in {{\textsf {\textit{results-at}}}}\,(x)} \mathfrak {C}(res) \\&\mathcal {E}(x) {:}= \min _{res\in {{\textsf {\textit{results-at}}}}\,(x)} {{\textsf {\textit{expansion}}}}\,(res) \\&\mathcal {E}^{-1}(x) {:}= \min _{res\in {{\textsf {\textit{results-at}}}}\,(x)} {{\textsf {\textit{preexpansion}}}}\,(res) \end{aligned}$$

One last property is ${{\textsf {\textit{invoke-nf}}}}\,$, which encodes if the numerical computations need to be interrupted and the results of the normal form need to be invoked. First, we define abstractly when this is necessary, namely on the *stable manifold* of the origin. That is, the set of all points, which tend to the origin in infinite time. We restrict our attention to the part of the stable manifold whose trajectories do not intersect ${\varSigma }$ for positive time.

#### Definition 15

$$\begin{aligned} {\varGamma }{:}= \left\{ x \mid [0, \infty ] \subseteq {{\textsf {\textit{ex-ivl}}}}\,~x \wedge (\forall t>0.~\phi (x, t) \notin {\varSigma }) \wedge (\phi (x, t) \longrightarrow _{t \rightarrow \infty } (0,0,0)) \right\} \end{aligned}$$

When ${{\textsf {\textit{invoke-nf}}}}\,$ is true, the computations will be interrupted once the reachable sets arrive at the small cube $L = [-0.1, 0.1]\times [-0.1, 0.1]\times [-0.1, 0.1]$ inside which the normal form estimates are valid. In our computations, solutions are guaranteed to enter the cube *L* through a rectangle *T* and the tangent vectors are in the cone that contains *DT*:

$$\begin{aligned} T {:}= ([-0.1, 0.1], [-0.00015, 0.00015], 0.1)\times \left( \begin{array}{c} [0.8, 1.7] \\ \, [0.0005, 0.002] \\ 0 \\ \end{array} \right) \end{aligned}$$

That is, sets are very slim in the *y*-direction, and the expanding direction is closely around the *x* axis. From Tucker’s analysis ([48, Proposition 3.1]), we devised the following bounds for the sets $E_1, E2$ (and corresponding cones in $DE_1, DE_2$) through which solutions emanating from *T* exit the cube *L*:

$$\begin{aligned} E_1 {:}= ([-0.12, -0.088],[-0.024, 0.024],[-0.012, 0.13])\times \left( \begin{array}{c} 0 \\ \, [-0.56, 0.56] \\ \, [-0.6, -0.08] \\ \end{array} \right) \end{aligned}$$

$$\begin{aligned} E_2 {:}= ([0.088, 0.12],[-0.024, 0.024],[-0.012, 0.13])\times \left( \begin{array}{c@{\quad }c@{\quad }c} 0 \\ \, [-0.56, 0.56] \\ \, [0.08, 0.6] \\ \end{array} \right) \end{aligned}$$

When we interrupt computations close to *L*, we check that the sets entering *L* do so within *T* and continuous computations from $E_1 \cup E_2$. Since we have not verified Tucker’s normal form theory, we need to trust the following assumption:

#### Assumption 14

(*Normal Form Theory Bounds*)

$$\begin{aligned} T \curvearrowright '_L (E_1 \cup E_2) \end{aligned}$$

### Checking the Input Data

In the previous section, we only defined what the ${{\textsf {\textit{input-data}}}}\,$ encodes. Now we check if the numerical bounds prescribed by the ${{\textsf {\textit{input-data}}}}\,$ are actually correct. This involves three steps: First, we need to find a suitable setup to be able to use the algorithm ${{\textsf {\textit{poincare}}}}\,$, which computes derivatives and not cones. Second, we set up the check that a single element of the ${{\textsf {\textit{input-data}}}}\,$ is correct. Third, we check all elements of the ${{\textsf {\textit{input-data}}}}\,$, from which we conclude the formal counterparts of Theorems 1 and 2.

#### Representation of Cones

Concerning the checking of cone conditions, first note that $\mathfrak {C}~res$ is an infinite cone, i.e., an unbounded set of vectors. In contrast to that, all of our numerical algorithms are tailored towards bounded enclosures. We therefore perform the computations with the line segment connecting the two tangent vectors with unit length. ${{\textsf {\textit{matrix-segment}}}}\,~x_1~y_1~x_2~y_2~e$ encodes a line segment (parameterized by *e*) in a matrix (such that it can be used as matrix initial condition *DX* of ${{\textsf {\textit{poincare}}}}\,$, compare Theorem 13). ${{\textsf {\textit{mat-seg-of-deg}}}}\,$ uses this to define the line segment between the endpoints of unit vectors with given angles *u*, *v* to the *x* axis. A cone can therefore represented with the help of ${{\textsf {\textit{mat-seg-of-deg}}}}\,$:

##### Lemma 2

(Matrix Representation of Cone)

$$\begin{aligned} \mathfrak {C}(res) = {{\textsf {\textit{cone}}}}\,~{{\textsf {\textit{hull}}}}\,~\left\{ \left( \begin{array}{c} m_{(1, 1)} \\ m_{(2, 1)} \\ 0 \end{array}\right) \Bigg | m \in {{\textsf {\textit{mat-seg-of-deg}}}}\,~({{\textsf {\textit{angle}}}}\,^{-}~res)~({{\textsf {\textit{angle}}}}\,^{+}~res) \right\} \end{aligned}$$

with

$$\begin{aligned}&{{\textsf {\textit{matrix-segment}}}}\,~x_1~y_1~x_2~y2~e{:}= \left( \begin{array}{c@{\quad }c@{\quad }c} x_1 + e \cdot (x_2 - x_1) &{} 0 &{} 0\\ y_1 + e \cdot (y_2 - y_1) &{} 0 &{} 0\\ 0 &{} 0 &{} 0 \end{array} \right) \\&{{\textsf {\textit{mat-seg-of-deg}}}}\,~u~v {:}= \\&\qquad {{\textsf {\textit{matrix-segment}}}}\,(\cos ~({{\textsf {\textit{rad}}}}\,~u)) (\sin ~({{\textsf {\textit{rad}}}}\,~u))(\cos ~({{\textsf {\textit{rad}}}}\,~v)) (\sin ~({{\textsf {\textit{rad}}}}\,~v)) [0, 1] \end{aligned}$$

#### Checking a Single Result Element


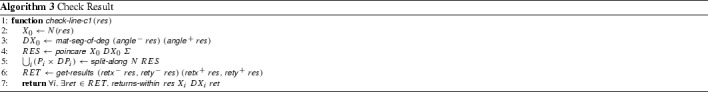


Algorithm 3 outlines how to check that a single result element $res \in {{\textsf {\textit{input-data}}}}\,$ represents correct numerical bounds. It works as follows: $X_0$ is the initial rectangle, $DX_0$ the initial data for the derivatives, which encodes the associated cone with angles ${{\textsf {\textit{angle}}}}\,^{-}~res$ and ${{\textsf {\textit{angle}}}}\,^{+}~res$. Then the ODE solver returns with a union of return images *RES*, which are split along the boundaries of the individual rectangles making up *N*. This splitting ensures that each individual element $(X_i, DX_i)$ resulting from the splitting is contained in exactly on individual element of *N*. We write singleton parts of the result of this splitting $X_i, DX_i$. In *RET*, there are all elements of the ${{\textsf {\textit{input-data}}}}\,$ within which *res* is specified to return. The final check makes sure that every part $X_i, DX_i$ of the splitting returns within one element *ret* of the collection *RET*. It is defined as follows and precisely formulates that *X* and *DX*, which emanate from a result *res* and hit the result *ret*, satisfy the prescribed bounds on cones and expansion.

$$\begin{aligned}&{{\textsf {\textit{returns-within}}}}\,~res~X~DX~ret{:}= \\&\qquad X \subseteq N(ret)~\wedge \\&\qquad {{\textsf {\textit{check-cone-bounds}}}}\,~({{\textsf {\textit{angle}}}}\,^{-}~res)~({{\textsf {\textit{angle}}}}\,^{+}~res)~X~DX~\wedge \\&\qquad \Vert DX\Vert \ge \mathcal {E}(res) \wedge \Vert DX\Vert \ge \mathcal {E}^{-1}(ret) \end{aligned}$$

${{\textsf {\textit{check-cone-bounds}}}}\,$ is checked using affine arithmetic: It assumes that $u_x$ and $u_y$ are on the line segment encoding a cone according to ${{\textsf {\textit{mat-seg-of-deg}}}}\,$, therefore checks that $u_z = 0$ and ignores the other entries of the argument matrix. It further checks that the segment is on the right side ($0 < u_x$) and that the boundary angles *L* and *U* (given in degrees) also represent a cone pointing to the right side. The main purpose is in the last line, the check that the angle of the vector $(u_x, u_y)$ with the horizontal axis is between *L* and *U*.

$$\begin{aligned}&{{\textsf {\textit{check-cone-bounds}}}}\,~L~U~ \left( \begin{array}{c} x \\ y \\ z \end{array} \right) ~ \left( \begin{array}{c@{\quad }c@{\quad }c} u_x &{} v_x &{} w_x \\ u_y &{} v_y &{} w_y \\ u_z &{} v_z &{} w_z \end{array} \right) {:}= \\&\qquad -90< L \wedge L \le U \wedge U< 90~\wedge \\&\qquad 0 < u_x \wedge u_z = 0~\wedge \\&\qquad \tan ({{\textsf {\textit{rad}}}}\,~L) \le \frac{u_y}{u_x} \wedge \frac{u_y}{u_x} \le \tan ({{\textsf {\textit{rad}}}}\,~U) \end{aligned}$$

Correctness of ${{\textsf {\textit{check-line-c1}}}}\,$ states that the set *N*(*res*) is mapped into the part ${{\textsf {\textit{return-of}}}}\,~res$ of the forward invariant set. Vectors in the cone $\mathfrak {C}(res)$ are mapped by the derivative $\mathsf {D}P$ into the cone field with the prescribed expansion estimates. The theorem states that the derivative exists and is defined when approaching *x* within ${\varSigma }_{\le }= \{(x, y, z)\mid z \le 27 \}$.

##### Theorem 15

(Correctness of *check-line-c1***)**

$$\begin{aligned}&{{\textsf {\textit{check-line-c1}}}}\,(res) = {{\textsf {\textit{return}}}}\,~{{\textsf {\textit{True}}}}\,~\implies \\&\quad \forall x \in N(res) - {\varGamma }.~\forall dx \in \mathfrak {C}(res).~ {{\textsf {\textit{returns-to}}}}\,~{\varSigma }~x \wedge P(x) \in N({{\textsf {\textit{return-of}}}}\,~res)~\wedge \\&\qquad (\exists DP.~(P~{{\textsf {\textit{has-derivative}}}}\,~DP)~({{\textsf {\textit{at}}}}\,~x~{{\textsf {\textit{within}}}}\,~{\varSigma }_{\le }) \\&\qquad \quad (\Vert DP(dx)\Vert \ge \mathcal {E}(res)\cdot \Vert dx\Vert )~\wedge \\&\qquad \quad (\exists ret \in {{\textsf {\textit{return-of}}}}\,~res. \\&\qquad \quad \quad P(x) \in N(ret)\wedge DP(dx) \in \mathfrak {C}(ret) \wedge \Vert DP(dx)\Vert \ge \mathcal {E}^{-1}(ret)\cdot \Vert dx\Vert )) \end{aligned}$$

The theorem follows rather directly from the definition of algorithm 3 and the specifications and definitions of the occurring functions.

#### Checking All Results

We have indeed the theorem that all ${{\textsf {\textit{input-data}}}}\,$ is correct:

##### Theorem 16

(Global Numerical Results)

$$\begin{aligned} \forall res\in {{\textsf {\textit{input-data}}}}\,.~{{\textsf {\textit{check-line-c1}}}}\,~res = {{\textsf {\textit{return}}}}\,~{{\textsf {\textit{True}}}}\,\end{aligned}$$

We prove formally that under the Assumption 14, Theorem 16 implies Theorems 1 and 2, which is the main result of this article. It follows from combining the individual instances of Theorem 15 in a suitable way.

Theorem 16 is proved by computing ${{\textsf {\textit{check-line-c1}}}}\,(res)$ for every $res \in {{\textsf {\textit{input-data}}}}\,$. The computations are carried out using by evaluating the statement

$$\begin{aligned} {{\textsf {\textit{Parallel.forall}}}}\,~(\lambda res.~{{\textsf {\textit{check-line-c1}}}}\,~res)~{{\textsf {\textit{input-data}}}}\,\end{aligned}$$

with Isabelle/HOL’s evaluation engine $\texttt {eval}$. ${{\textsf {\textit{Parallel.forall}}}}\,$ results in parallel processing of the 400 individual elements of ${{\textsf {\textit{input-data}}}}\,$. Further parallelism is introduced when enclosures are split during reachability analysis. Split sets can be processed in parallel until they reach the next (intermediate) Poincaré section, where they might be (partially merged) upon resolving the intersection (Sect. 6.5).

**Fig. 11:**
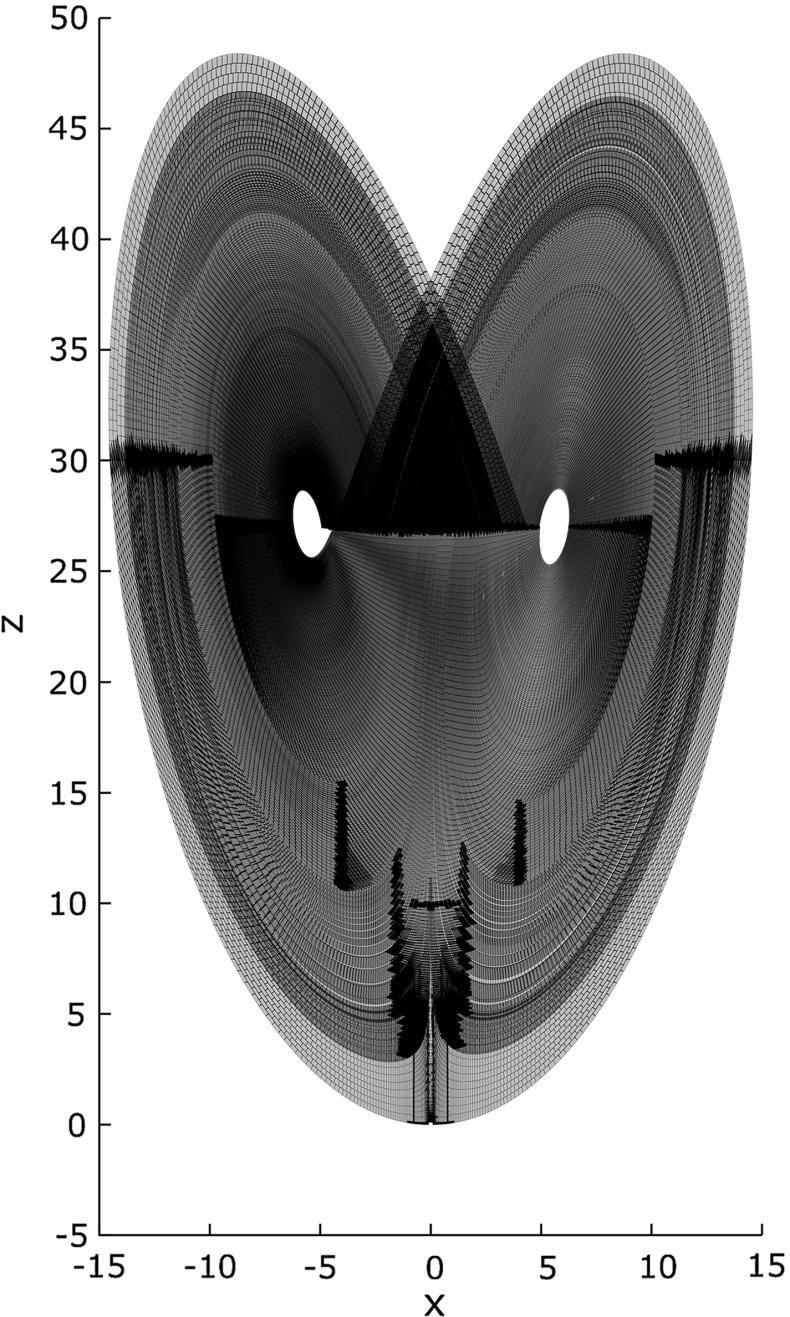
Enclosure of $\phi \{(x, [0; \tau (x)]) \mid x \in N^+\}$, during the computation of $P(N^+)$

Figure 11 shows the plot of an enclosure for the Lorenz attractor resulting from the verified computation. The plot hints at the intermediate Poincaré sections that were manually set up (for some initial rectangles) at about $z=27$, $z=30$, $x=\pm 5$, $x=\pm 1.5$, $x=\pm 1$, $x=\pm 0.75$, $x=\pm 0.1$, and $z=0.1$. The black part of Fig. 10 is an enclosure for $P(N^+)$ resulting from these computations, and it is as expected and verified contained in *N*.

The timing results of a computation on a machine with 22 cores^4^ are given below:

- Threads: 22
- Elapsed Time: 6 h 33 min 9 s
- CPU Time: 131 h 52 min 40 s
- Parallelization Factor: 20.13
- Garbage Collection Time: 42 min 36 s

To compare this with Tucker’s C${++}$ program, I compiled Tucker’s program in a Virtual Machine running Ubuntu 4.20 and gcc version 3.3.4 on a machine with a 2,6 GHz Intel® Core™ i7 CPU and 16 GB RAM. Tucker’s program finished after a total CPU time of 30h and 24min. The algorithms and data structures are very different, so a direct comparison is hard. But with regard to the total CPU time (131 h) of our algorithm, a factor of less than five compared to a C${++}$ program signifies reasonable performance for a verified algorithm.

In earlier developments [20], an enclosure for the Lorenz attractor was computed with neither derivative nor cones. This earlier version verified an enclosure for the Lorenz attractor in about 7000 CPU hours. With the present version algorithms, such a computation (without derivatives and cones) can be performed in about 3 CPU hours. The speedup compared to the earlier work is mostly due to less aggressive splitting of reachable sets, and a smaller number of intermediate Poincaré sections: In the earlier work [20], intermediate Poincaré sections were introduced heuristically on-the-fly, and in the present work only where they are really effective. This is beneficial, because resolving the intersection incurs overapproximations.

## Conclusion

This article presented an overview of the diversity of algorithms, abstract results and techniques for the formal verification of a general-purpose ODE solver and its application to the rigorous numerical part of Tucker’s proof.

### Future Work

Future work would be to lift the assumption 14 on the normal form theory to formal foundations. This involves in particular multivariate formal power series and a number of analytic estimations, proving existence and convergence of specifically constructed formal power series. This involves more than 24 pages of Tucker’s article and is therefore a larger formalization effort. It also includes computer assisted parts: smalldiv.cc and coeffs.cc help devising the normal form, but neither their specification nor their implementation is particularly challenging; they essentially only evaluate a large number of fixed arithmetic expressions.

Another direction for future work would be to formally conclude from the numerical results that the Lorenz equations support a robust singular hyperbolic attractor. One part would be to prove (similar to Tucker’s expansion.cc) that the expansion estimates given by $\mathcal {E}$ are sufficiently large to guarantee the *locally eventually onto* property. We validated this with a non-verified computation for the expansion estimates prescribed by our ${{\textsf {\textit{input-data}}}}\,$. Further mathematical foundations are required in order to conclude from the computed forward invariant conefield $\mathfrak {C}$ and the expansion bounds that there exists a stable foliation, and that this foliation can be used to reduce the two-dimensional dynamics on ${\varSigma }$ to a one-dimensional map. This requires in particular the formalization of differentiable manifolds, and theorems like the existence of differentiable invariant sections for fiber contractions [41].

### Size of Code

Table 1 shows some statistics on the size in terms of lines of code of several programs related to this verification. RODES is the rigorous ODE solver used by Tucker, it consists of 3800 lines of C${++}$ code and builds on a library for interval arithmetic (Profil/BIAS) of about twice the size. Similar to the sum of those two is the size of the generated SML code. The verification required more effort, but the largest part is generic: the part specific to the Lorenz attractor makes up less than 10% of the total number of lines of code.

**Table 1:** Size of code and formalization

	Sections	Language	Lines of code/proof
RODES		C${++}$	3800
Profil/BIAS		C${++}$	8852
generated ODE solver		SML	13200
Flow, Poincaré map	2	Isabelle/HOL theory	12000–16500
Affine Arithmetic	3		8500
Intersection	4		5000
Refinement/Enclosures	3		5000
Reachability Analysis	6		10000
Lorenz Attractor	7		3000

### Trust Base

We use the evaluation oracle eval in Isabelle/HOL. This is common practice to speed up rewriting. Isabelle/HOL equations are mapped to function definitions in Standard ML. These are compiled and evaluated in PolyML.^5^ We also use the common extension (HOL-Library.Code_Target_Numeral in Isabelle2017) of the code generator that maps Isabelle/HOL integers to the integer type IntInf.int of PolyML, which can be based on either PolyML’s own implementation of arbitrary precision integers or GMP [8].

This setup means that the trusted code base is increased: The translation of Isabelle/HOL terms to SML code is not verified. One needs to trust PolyML and its compiler, but PolyML is Isabelle’s implementation language and therefore anyhow part of the trusted code base.

Reducing the trusted code base is an orthogonal issue: there is ongoing work [16, 17] for verified code generation to CakeML [28], a verified implementation of ML.

Isabelle/HOL’s eval speeds up evaluation by translating terms to the implementation language of the proof checker (PolyML). In view of this, it is more similar to Coq’s native_compute [2], which evaluates terms after translation to Coq’s implementation language OCaml, than to Coq’s virtual machine [9].

### Related Work

*Integrals and Differential Equations in Proof Assistants* Spitters and Makarov [33] implement Picard iteration to calculate solutions of ODEs in the interactive theorem prover Coq, but restricted to relatively short existence intervals. Boldo et al. [3] approximate the solution of one particular partial differential equation with a C-program and verify its correctness in Coq. Mahboubi and Sibut-Pinote [32] compute rigorous approximations of integrals with Taylor models.

*Rigorous Numerics in Proof Assistants* Rigorous numerical approximation of arithmetic expressions has been done in Coq [34] for different types of enclosures (Taylor models, intervals, centered forms). Muñoz and Lester [37] use rational interval arithmetic in PVS. Rigorous numerics with first order interval approximations has been implemented by Solovyev for the Flyspeck project [44] in HOL Light. This work is remarkable in that it is not relying on code generation but uses only primitive inference rules of HOL Light’s kernel.

*Computational Geometry* Pichardie and Bertot [40] were the first to formalize the ccw system and verify a functional convex hull algorithm in Coq. Meikle and Fleuriot [35] formalized an imperative algorithm and verified it using Hoare logic in Isabelle/HOL. Brun et al. [5] verify an algorithm based on hypermaps to compute the convex hull.

## Footnotes

1 That is, Lorenz’ original equations for the classical parameters $\beta = \frac{8}{3}, \sigma =10, \rho = 28$ in Jordan normal form using $\tau {:}= \sqrt{(\sigma + 1)^2 + 4 \sigma (\rho - 1)}$, $k_1 {:}= \frac{\sigma }{\tau }$, $k_2 {:}= \frac{\sigma - 1 + \tau }{2 \sigma }$, $k_3 {:}= \frac{\sigma - 1 - \tau }{2 \sigma }$, $\lambda _1 {:}= \frac{- \sigma - 1 + \tau }{2}$, $\lambda _2 {:}= \frac{- \sigma - 1 - \tau }{2}$, and $\lambda _3 {:}= - \beta$.

2 Vectors of length *n* are represented by a type of functions $\iota \rightarrow \alpha$, where *n* equals the cardinality of the finite type $\iota$.

3 http://www2.math.uu.se/~warwick/main/rodes/ResultFile.

4 Intel$\circledR$ Xeon$\circledR$ CPU E5-2699 v4 @ 2.20 GHz.

5 http://polyml.org/.
